# Oncostatin M Reduces Pathological Neovascularization in the Retina Through Müller Cell Activation

**DOI:** 10.1167/iovs.65.1.22

**Published:** 2024-01-08

**Authors:** Julian Rapp, Alban Hospach, Paula Liang, Melanie Schwämmle, Lisa Renz, Hansjürgen Agostini, Günther Schlunck, Felicitas Bucher

**Affiliations:** 1 Eye Center, Medical Center – University of Freiburg, Faculty of Medicine, University of Freiburg, Freiburg, Germany; 2 Faculty of Biology, University of Freiburg, Freiburg, Germany

**Keywords:** OSM, OIR, neovascularization, retina, glia

## Abstract

**Purpose:**

Continuous vision loss due to vasoproliferative eye disease still represents an unsolved issue despite anti-vascular endothelial growth factor (VEGF) therapy. The impact of signal transducer and activator of transcription 3 (STAT3) signaling on retinal angiogenesis and its potential use as a therapeutic target remain controversial. In vitro, oncostatin M (OSM), as a strong STAT3 activator, possesses robust proangiogenic activity. This study investigated to what extent the proangiogenic effects of OSM translate to the in vivo setting of vasoproliferative eye disease.

**Methods:**

The in vitro effect of OSM on endothelial cells was investigated in the spheroid sprouting assay and through RNA sequencing. The mouse model for oxygen-induced retinopathy (OIR) was used to evaluate the impact of OSM in vivo. Signaling patterns were measured by western blot and retinal cryosections. Primary Müller cell cultures were used to evaluate the effect of OSM on the Müller cell secretome. Murine retinal vascular endothelial cells were isolated from OIR retinas using fluorescence-activated cell sorting (FACS) and were used for RNA sequencing.

**Results:**

Although OSM induced pro-angiogenic responses in vitro, in the OIR model intravitreal injection of OSM reduced retinal neovascularization by 65.2% and vaso-obliteration by 45.5% in Müller cells. Injecting OSM into the vitreous activated the STAT3 signaling pathway in multiple retinal cell types, including Müller cells. In vitro, OSM treatment increased CXCL10 secretion. RNA sequencing of sorted vascular endothelial cells at OIR P17 following OSM treatment indicated downregulation of angiogenesis- and mitosis-associated genes.

**Conclusions:**

In vivo, OSM reveals a beneficial angiomodulatory effect by activating Müller cells and changing their secretome. The data highlight contradictions between cytokine-induced effects in vitro and in vivo depending on the cell types mediating the effect.

Major causes of vision loss in the developed world are vasoproliferative eye diseases such as diabetic retinopathy (DR) or neovascular age-related macular degeneration (nAMD).^1^ A milestone in treating these diseases was the establishment of intravitreal injections of antibodies against the vascular endothelial growth factor (VEGF).^2^^,^^3^ Nevertheless, non-responders and long-term disease progression under anti-VEGF therapy still represent major limitations.^4^^–^^6^ Other rare forms of retinal eye diseases with secondary vascular alterations do not have approved treatment options as is the case for macular telangiectasia 1 or 2.^7^ New therapeutic concepts therefore seem necessary to further decrease the risk of vision loss in those patients.

Both DR and nAMD are partly driven by an inflammatory microenvironment that ultimately adds to the development of retinal neovascularization.^8^^–^^10^ Modulating the inflammatory microenvironment through cytokines or cytokine-targeting antibodies to beneficially impact the retinal vascular phenotype represents a novel therapeutic approach. Antibodies targeting interleukin 6 (IL-6) are currently being evaluated in clinical trials for the treatment of uveitic macular edema and are discussed for their therapeutic potential in macular edema due to retinal neovascular disease.^11^

The IL-6 cytokine family represents a heterogeneous class of inflammatory and pleiotropic cytokines including IL-6, IL-11, IL-27, leukemia inhibitory factor (LIF), cardiotrophin-1 (CT-1), cardiotrophin-like cytokine (CLC), ciliary neurotrophic factor (CNTF), and oncostatin M (OSM), which share similar receptor complexes and the signal transducer and activator of transcription 3 (STAT3) signaling pathway as a common intracellular signaling pattern.^12^ In our previous work, we reported a strong angiomodulatory effect of CNTF-induced STAT3 signaling in various mouse models of vasoproliferative eye diseases which was based on a direct anti-angiogenic effect on vascular endothelial cells as well as an indirect, Müller cell–mediated effect.^13^^,^^14^

OSM represents another well-characterized member of the IL-6 cytokine family. In contrast to CNTF, it is known for its strong pro-angiogenic effects on vascular endothelial cells despite sharing crucial receptor and signaling components including gp130 and STAT3.^15^^–^^17^ Our own in vitro studies revealed that OSM and CNTF induced similar transcriptomic changes in vascular endothelial cells, but only OSM strongly enhanced their metabolic activity to fuel endothelial cell proliferation, migration, and sprouting.^17^ In humans, OSM binds to a heterodimeric signaling complex of gp130 and OSM receptor-beta (OSMRβ) or leukemia inhibiting factor receptor (LIFR),^18^ whereas for mice murine OSM only signals through its specific receptor and not LIFR.^19^ Next to the STAT family, particularly STAT3 but also STAT1 and STAT5, OSM activates the ERK and AKT cascades.^20^^,^^21^

OSM has repeatedly been linked to cancer progression and pathological angiogenesis outside the eye.^22^^,^^23^ In the eye, the expression pattern of OSMR has not yet been fully characterized. OSM receptor was reported to be expressed by photoreceptors, and treatment with OSM led to regeneration of rods and cones.^24^ However, its impact on retinal angiogenesis and retinal vasoproliferative disease remains unclear. Based on the pro-angiogenic effects of OSM on vascular endothelial cells in vitro, we wanted to evaluate whether the pro-angiogenic effect of OSM translates into in vivo settings and fuels the development of neovascularization in retinal disease.

In this study, we detected a beneficial angiomodulatory effect of OSM after its intravitreal injection in the oxygen-induced retinopathy (OIR) model. OSM induced STAT3 signaling in multiple retinal cell types, including Müller cells and vascular endothelial cells. In vitro experiments suggest that OSM altered the Müller cell secretome. RNA sequencing on isolated retinal vascular endothelial cells from OIR P17 mice portrayed the transcriptomic shift in these cells as a result of OSM treatment at P12. These new insights emphasize the importance of Müller cells in the context of vasoproliferative eye disease, which can result in substantial disparities between the effects of angiogenic agents in vitro and in vivo.

## Methods

### HUVEC Cell Culture

Human umbilical vein endothelial cells (HUVECS, #C2519A; Lonza, Basel, Switzerland) were grown in EBM-2 Endothelial Cell Growth Basal Medium-2 (EGM-2, #CC-3162; Lonza). HUVECs of the third passage dissolved in 80% EGM/10% fetal bovine serum (FBS, #S0615; Biochrom, Berlin, Germany) and 10% dimethyl sulfoxide (#472301; Sigma-Aldrich, St. Louis, MO, USA) were frozen in liquid nitrogen for storage. For all experiments, only HUVECs of passage 6 were used. RNA and protein samples were generated by seeding 150,000 HUVECs in six-well plates in EGM and incubating them overnight. On the next day, cells were stimulated by 100 ng/mL OSM (#300-100; PeproTech, Cranbury, NJ, USA) plus 25 ng/mL VEGF (#100-20; PeproTech) or just 25 ng/mL VEGF in EBM-2 Endothelial Cell Growth Basal Medium-2 (#CC-3156; Lonza) supplemented with 2% FBS (#FBS. S 0615; Bio&SELL, Feucht, Germany) for 24 hours. Cells were lysed in 700 µL ice-cold QIAzol (#79306; QIAGEN, Hilden, Germany) incubated for 5 minutes while proteins were collected by lysing cells in ice-cold 150 µL T-PER Tissue Protein Extraction Reagent (#78510; Thermo Fisher Scientific, Waltham, MA, USA) supplemented by 1:100 protease inhibitors (#78420; Thermo Fisher Scientific) and 1:100 phosphatase inhibitor (#87786; Thermo Fisher Scientific). Protein samples were centrifuged at 12.000 relative centrifugal force (RCF), and the supernatant was used for downstream applications.

### HRMVEC Cell Culture

Human microvascular endothelial cells (HRMVECs, #ACBRI 181; Cell Systems, Kirkland, WA, USA) were grown in HRMVEC medium (#PB-MH-100-4090; PELOBiotech, Planegg, Germany) supplemented with 10% FBS. For all experiments only HRMVECs of passage 9 were used. For RNA harvest, 75,000 cells were seeded in in 12-well plates and incubated overnight. Cytokines for stimulation were diluted in HRMVEC medium without any supplement but 6% FBS, and cells were stimulated for 24 hours. The same cytokine concentrations as in HUVEC cell culture were used, and downstream analysis was performed as described for HUVECs.

### Primary Müller Cell Culture

Isolation of murine Müller cells for in vitro experiments followed established techniques.^14^^,^^25^ A papain dissociation system (#LK003150; Worthington Biochemical Corporation, Lakewood, NJ, USA) was used to digest whole retinas of P11 C57BL/6J mice free of the retinal degeneration 1 (RD1) mutation and RD8 mutation that were provided by Charles River Laboratories (Wilmington, MA, USA). Primary Müller cells were grown in Neurobasal-A medium (#10888022; Thermo Fisher Scientific), N-2 Supplement (#17502048; Thermo Fisher Scientific), 10% FBS, 2-mM l-glutamine (#25030-081; Thermo Fisher Scientific), and Merck penicillin/streptomycin (#P4333; Sigma-Aldrich) containing 100 ng/mL epidermal growth factor (#PHG0311; Thermo Fisher Scientific). After expansion including two passages, the medium was changed to a differentiation medium containing Neurobasal-A medium, 1% FBS, N-2 Supplement, 2-mM l-glutamine, penicillin/streptomycin, and 50× B-27 Supplement (#17504044; Thermo Fisher Scientific) for 7 days. Cells were stimulated with 100 ng/mL murine OSM (#495-MO-025; R&D Systems, Minneapolis, MN, USA) in differentiation media for 15 minutes and lysed by T-PER Tissue Protein Extraction Reagent including 1:100 protease inhibitor and 1:100 phosphatase inhibitor. Müller cell supernatant was generated by stimulating cells with 100 ng/mL OSM or PBS control in differentiation media, changing the medium after 15 minutes and collecting the medium after 72 hours of conditioning. Supernatant was evaluated for protein expression by the Proteome Profiler Mouse Angiogenesis Array Kit (#ARY015; R&D Systems). All targets of the profiler and their respective coordinates can be found in Supplementary Table S1. Statistical analysis was run on three biological replicates. Figures show representative images of profiler membranes. The other two samples can be found in Supplementary Figure S5.

### Spheroid Sprouting Assay

The spheroid sprouting assay was performed as previously published.^17^ In brief, HUVECs in EGM-2 supplemented with 0.25% carboxymethylcellulose (#M0512; Sigma-Aldrich) formed hanging drops overnight. Spheroids were seeded in a three-dimensional collagen matrix consisting of 50% rat tail collagen (#354236; Corning, Corning, NY, USA), 48% EBM containing 0.25% carboxymethylcellulose, and 2% FBS. The final matrix was buffered by adding 0.035% 1-M HEPES buffer (#P05-01100; PAN-Biotech, Aidenbach. Germany) of the final volume and stimulated by 25-ng/mL VEGF or 100-ng/mL OSM together with 25-ng/mL VEGF. On the next day, phase-contrast images of all spheroids were taken with an inverse microscope (ZEISS Axiovert A1; Carl Zeiss Microscopy, Jena, Germany). The measuring tool of ImageJ Fiji was used to determine sprout lengths. The relative sprouting length (RSL) was calculated as the average of the cumulative sprouting length per spheroid normalized to the control groups.

### Animal Models

The previously published mouse model of oxygen-induced retinopathy (OIR)^26^ using C57BL/6J mice provided by Charles River Laboratories or ALDH1L1-GFP^+^ transgenic mice (The Jackson Laboratory, Bar Harbor, ME, USA), both free of RD1 and RD8 mutations, were used for in vivo experiments. All animals were treated in adherence to the ARVO Statement for the Use of Animals in Ophthalmic and Vision research, and all experimental setups were reviewed and approved by the local animal welfare committee (G20/44, G22/53). Intravitreal injections of 0.5 µL of 100-ng/µL OSM (#495-MO-025; R&D Systems) or PBS (#14190-094; Thermo Fisher Scientific) were conducted at postnatal day 12 (P12) using a sharp 33.5-gauge Hamilton syringe after eyelids were carefully opened with blunt forceps. The contralateral eyes were used for PBS control injections.

#### Whole Retina Protein Lysates for Signaling Analysis

Whole retinas were lysed in 120 µL T-PER Tissue Protein Extraction Reagent per retina supplemented by protease inhibitors and phosphatase inhibitors centrifuged at 12,000 RCF, and supernatant was used for downstream applications.

#### Retinal Flatmounts

Eyes were fixed in 4% paraformaldehyde (PFA) for 40 minutes at 4°C. Retinal wholemounts were dissected and stained in 1:200 Griffonia Simplicifolia Lectin 1 (GSL 1) Isolectin B4 (#FL-1201-.5; Vector Laboratories, Newark, CA, USA) overnight at 4°C. Stained retinas were flattened by four cuts to create a cloverleaf shape and mounted on slides in mounting medium (#S302380-2; Agilent Technologies, Santa Clara, CA, USA). Slides were imaged with a NanoZoomer S60 Digital slide scanner (#C13210-04; Hamamatsu Photonics, Shizuoka, Japan). The areas of neovascularization (NV) and vaso-obliteration (VO) in relation to the total flatmount area were quantified according to established protocols.^27^

#### Retinal Cryosections for Immunohistochemistry

Eyes were fixed for 1 hour in 2% PFA at 4°C followed by a 24-hour incubation period at 4°C in 20% sucrose (#4621.1; Carl Roth, Karlsruhe, Germany). Samples were embedded and frozen in O.C.T (#4583; Sakura Finetek, Torrance, CA, USA). Then, 10-µm sections were fixed in ice-cold Merck acetone (#32201; Sigma-Aldrich) for 10 minutes, permeabilized in 0.3% Triton X-100 (#T8787, Sigma-Aldrich) for 20 minutes, and blocked in 10% normal goat serum (#JAC-005-000-121; Jackson ImmunoResearch, West Grove, PA, USA) in PBS for 1 hour. Sections were stained overnight at 4°C, followed by respective fluorophore-tagged secondary antibodies at room temperature for 1 hour. All antibodies and their dilutions can be found in Supplementary Table S2. Staining was finalized by a 20-minute treatment with 1:200 Griffonia (Bandeiraea) Simplicifolia Lectin I (#RL-1102-2; Vector Laboratories) and 1:1000 4′,6-diamidino-2-phenylindole (DAPI, #MBD0015; Sigma-Aldrich) 10-minute incubation. A TCS SP8 confocal microscope (Leica, Wetzlar, Germany) and LASX 3.5.7 software were used to generate high-quality representative images.

### Western Blot

Protein lysates were diluted in Laemmli Buffer (#1610747; Bio-Rad Laboratories, Hercules, CA, USA) and mercaptoethanol (#M3148; Sigma-Aldrich) at a 0.75/0.225/0.025 ratio and transferred to a Immobilon-P PVDF Membrane (#IPVH00010; EMD Millipore, Burlington, MA, USA) after gel electrophoresis. After 30-minute blocking of membranes with 3% bovine serum albumin, primary antibodies were incubated overnight. Respective secondary antibodies were incubated for 1 hour at room temperature. All antibodies and their dilutions can be found in Supplementary Table S2. A Fusion FX system (Vilber, Collégien, France) visualized bands by enhanced chemiluminescence (#RPN2232; GE Healthcare, Chicago, IL, USA). For semiquantitative analysis, the gel analyzer platform of ImageJ Fiji was used, and readouts were normalized to the respective GAPDH controls. Statistical analyses were always run on three biological replicates from three independent experiments. Figures show representative images of western blots. Uncut blots can be found in Supplementary Figure S5.

### Reverse Transcription–Polymerase Chain Reaction and Quantitative Polymerase Chain Reaction

RNA was extracted by following the miRNAeasy Mini Kit (#217004; QIAGEN), and reverse transcription was conducted using the SuperScript IV Reverse Transcriptase (#18090-010; Thermo Fisher Scientific) on a C1000 Touch Thermal Cycler (#1851197; Bio-Rad Laboratories). Polymerase chain reaction (PCR) and gel electrophoresis were conducted with a master mix consisting of 15 µL nuclease-free water (#129117; QIAGEN), 5 µL reaction buffer (#M7918; Promega, Madison, WI, USA), 0.4 µL deoxynucleotide triphosphate (dNTP, #R0192; Thermo Fisher Scientific), 0.1 µL GoTaq DNA Polymerase (#M7408; Promega), 5 µM of the specific primer, and 2 µL cDNA. All primers can be found in Supplementary Table S3. The C1000 Touch Thermal Cycler ran the PCR, and products were analyzed on a 2% agarose gel by visualizing bands with GelRed (1:100, #41003; VWR, Radnor, PA, USA). The GeneRuler 50 bp DNA Ladder (#SM0373; Thermo Fisher Scientific) helped to determine product sizes. For quantitative PCR (qPCR), instructions for a SYBR Green kit (#RR420A; Takara Bio, Shiga, Japan) were followed, and a LightCycler 480 (Roche Diagnostics, Indianapolis, IN, USA) measured data. All primers can be found in Supplementary Table S3. Data were analyzed using the 2^−ΔΔCq^ method.^28^

### Isolation of Retinal Vascular Endothelial Cells From Whole Retinas

Murine retinal vascular endothelial cells were isolated by applying a modified version of published protocols.^29^^,^^30^ At P17, two retinas of one mouse were pooled per sample, and a single cell suspension was obtained by digestion (45 minutes at 37°C) in 0.75 mL Hanks’ Balanced Salt Solution (#14025092; Thermo Fisher Scientific) containing 1.6 mg/mL Liberase DL (#5466202001; Roche Diagnostics) and 0.1 mg/ml DNase (#10104159001; Roche Diagnostics). Clumps were removed by passing the suspension through a 50-µm cell strainer (#04-004-2327; Sysmex, Kobe, Japan). Dead cells were labeled by 5-minute DAPI (1:10,000) staining. Following a 20-minutes Fc block by CD16/CD32 antibodies (#553141; Becton Dickinson, Franklin Lakes, NJ, USA), cells were stained with CD31 (#12-0211-82; Thermo Fisher Scientific) and CD45 monoclonal antibody (#56-0451-82; Thermo Fisher Scientific) for 20 minutes at 4°C. All antibodies and their dilutions can be found in Supplementary Table S2. Retinal vascular endothelial cells were defined in the sorting process by omitting clumps and debris by side scatter area (SSC-A)/forward scatter area (FSC-A) gating, excluding doublets by a SSC-width (W)/FSC-W gate and DAPI^+^ dead cells while sorting CD31^+^/CD45^–^ events from this population. Cells were sorted in 100 µL RNAprotect (76526; QIAGEN) by a MoFlo Astrios cell sorter (Beckman Coulter, Brea, CA, USA)

### RNA Sequencing

The Center of Excellence for Fluorescent Bioanalytics (University of Regensburg, Regensburg, Germany; www.kfb-regensburg.de) conducted RNA extraction followed by library preparation and sequencing. The RNA of in vivo samples was isolated according to the RNeasy Plus Micro Kit (74034; QIAGEN). Using 500 pg total RNA, the SMARTer Ultra Low Input RNA Kit for Sequencing v4 (#634893; Takara Bio) including polyA priming was used to generate first-strand cDNA, which was amplified by long-distance (LD) PCR (12 cycles) and purified via magnetic bead clean-up. Libraries were prepared according to the Nextera XT DNA Library Prep Kit (#FC-131-1024; Illumina, San Diego, CA, USA). Equimolar amounts of each library were sequenced on an Illumina NextSeq 2000 instrument controlled by NextSeq 2000 Control Software (NCS) 1.2.0.36376, using one 50-cycle P3 Flow Cell with the dual-index, single-read (SR) run parameter. The RNA Integrity Numbers (RIN), the targeted sequencing depth, and sequencing depth for all samples can be found in Supplementary Tables S4 and S5.

### Bioinformatics

In vitro data were accessed from Gene Expression Omnibus under GSE198484.^17^ Raw FASTQ files were uploaded to Galaxy.eu (https://usegalaxy.eu)^31^ and checked for quality using FASTQC (Galaxy Version 0.72). Due to very good quality of reads, no further preprocessing was necessary. The data were aligned to the murine (GRCm39) or human (GRCh38) reference genome provided by GENCODE (www.gencodegenes.org, downloaded September 2021) by the STAR aligner (Galaxy Version 2.7.8a).^32^ Aligned reads were assigned to genes by featureCounts (Galaxy Version 2.0.1)^33^ using the gene annotations from GENCODE (downloaded September 2021). Standard settings were applied for all steps. Counts were further analyzed in R 4.0.2 (R Foundation for Statistical Computing, Vienna, Austria) by the DESeq2 package^34^ including a correction for batch effects using the removeBatchEffect function from the limma R package.^35^ Differentially expressed genes (DEGs) were defined by *P*_adj_ < 0.05. Ensembl 101 IDs were matched to Mouse Genome Informatics (MGI) gene symbols and the equivalent HUGO Gene Nomenclature Committee (HGNC) symbols using biomaRt for R^36^ for in vivo samples. Gene Ontology (GO) term enrichment analysis was performed using the clusterProfiler package for R.^37^ For gene set enrichment analysis (GSEA), genes with a mean count > 10 were ranked according to their log_2_ fold change after using established log_2_ fold change shrinkage methods of the DESeq2 package in normal settings. The fgsea package^38^ calculated enriched gene sets, which were downloaded from MsigDB (http://www.gsea-msigdb.org/gsea/msigdb) in April 2021. The packages ggplot2^39^ and ComplexHeatmaps^40^ supported visualization.

### Statistics

If not otherwise stated, a Mann–Whitney test was used for assessing statistical significance. *P* < 0.05 was considered significant and is indicated by an asterisk; otherwise, **P < 0.01, ***P < 0.001, and ****P < 0.0001. Graphs visualize the means and error bars the standard error of the mean (SEM) if not stated otherwise. The dataset generated in this study can be found on Gene Expression Omnibus (GSE227350).

## Results

### OSM Induces Angiogenesis In Vitro and Elicits an Inflammatory Transcriptomic Response in Vascular Endothelial Cells

In our previous work, we observed that OSM, in contrast to CNTF, has a strong pro-angiogenic effect on vascular endothelial cells in in vitro angiogenesis assays despite sharing STAT3 as common intracellular signaling pathway.^17^ We also showed that the anti-angiogenic effect of CNTF translated into in vivo mouse models of vasoproliferative eye diseases by activating Müller cells.^13^^,^^14^ In the current study, we focused on OSM to evaluate whether its direct pro-angiogenic impact on vascular endothelial cells in vitro would translate in an in vivo setting as well. Alternatively, we sought to determine if OSM might elicit a different response following the pattern of CNTF in vivo.

**Figure 1.**
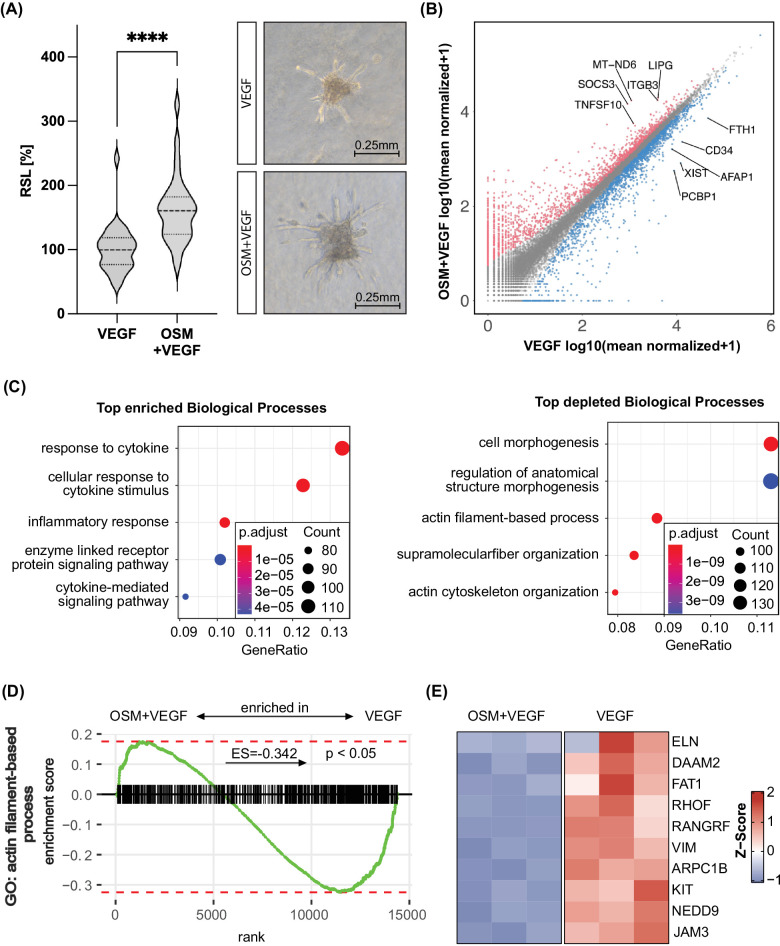
OSM exerted a pro-angiogenic and pro-inflammatory influence on endothelial cells. (**A**) Violin plot of the RSL in the spheroid sprouting assays of HUVECs stimulated for 18 hours by OSM+VEGF or VEGF. Medians are visualized by *dashed lines* and quartiles by *dotted lines*. The data incorporate the data from three independent experiments with 8 to 12 spheroids each. (**B**) Scatterplot of total counts in the RNA sequencing data of HUVECs stimulated by OSM+VEGF or VEGF for 24 hours. Genes considered to be DEGs and upregulated are indicated in *red*, and downregulated DEGs are *blue*. The highest five expressed DEGs with an absolute log_2_ fold change > 2 in each category are labeled. (**C**) GO term enrichment analysis for biological processes using DEGs between OSM+VEGF- and VEGF-stimulated HUVECs for 24 hours. Enrichment refers to enhanced expression in OSM+VEGF-treated samples and depletion to reduced expression in OSM+VEGF samples. Visualized are the five most enriched GO terms according to their respective adjusted *P* values; they are ranked according to their share of the total number of DEGs (GeneRatio). (**D**) GSEA of RNA sequencing data for the GO term “actin filament-based process” (GO:0030029). A positive enrichment score refers to enrichment of the specific set for OSM+VEGF-incubated HUVECs in contrast to cells just stimulated by VEGF. (**E**) Heatmap of the first 10 leading-edge genes of the GSEA from (**D**) using the *Z*-score.

In a first step, we used the spheroid sprouting assays as functional angiogenesis assays and confirmed that OSM enhanced VEGF-induced sprouting by 62% on average (Fig. 1A). On a transcriptomic level, HUVECs co-stimulated with OSM and VEGF showed a clear shift in the transcriptome compared to cells exposed to VEGF alone, represented by a good separation in principal component analysis (PCA) (Supplementary Fig. S1A). In total, 1707 genes were considered upregulated by the OSM+VEGF treatment, and 1622 were downregulated (Fig. 1B); 1950 DEGs had an absolute log_2_ fold change > 1. All DEGs, including their log_2_ fold changes and adjusted *P* values can be found in Supplementary File S1. Using a GO term enrichment analysis for biological processes to screen for patterns in the transcriptomic changes, various inflammation-associated GO terms were enriched significantly for the OSM+VEGF-treated samples, as indicated by “inflammatory response” (GO:0006954) ranking among the top five most enriched terms (Fig. 1C). Prominent downregulated terms were “cell morphogenesis,” “actin filament-based process” (GO:0030029), and “actin cytoskeleton organization” (GO:0030036) (Fig 1C). More details on the DEGs linked to this enrichment analysis can be found in Supplementary Tables S6 and S7. Surprisingly, the GO term “angiogenesis” (GO:0001525) was also depleted in response to OSM treatment in GSEA (Supplementary Fig. S1B). As OSM is known to signal through the STAT3 and ERK pathway,^17^ we then investigated whether a gene signature of these pathways could be observed in the RNA sequencing dataset. In GSEA, the hallmark STAT3 gene set showed pronounced enrichment for OSM+VEGF-treated samples (Supplementary Fig. S1C), with high expression of typical STAT3 downstream targets in the leading-edge genes such as *SOCS3* (Supplementary Fig. S1D). In contrast, the ERK signaling pathway and its downstream targets were not significantly enriched in the OSM+VEGF group represented by a GSEA for “ERK1 and ERK2 cascade” (GO:0070371), stressing the importance of STAT3 in the signaling process (Supplementary Fig. S1E). To further validate these data, we performed qPCR with selected DEGs which are also known STAT3 targets. *SOCS3* and *LIPG*, which were defined in the RNA sequencing as DEGs, were also significantly altered in their expression level in classic qPCR (Supplemental Fig. S1F), giving us confidence in the gathered data and the role of STAT3 in OSM signal transduction. qPCR data for the same targets in HRMVECs revealed similar tendencies, suggesting that observed changes in gene expression also translate to microvascular endothelial cell types (Supplementary Fig. S1F).

As “actin cytoskeleton organization” (GO:0030036) and other cytoskeleton-related GO terms were depleted in the analysis, we further explored the impact of OSM on this important biological process for angiogenesis. GSEA further confirmed the ability of OSM to act on the actin filament system on a transcriptomic level, as a significant depletion was noted for “actin filament-based process” (GO:0030029; enrichment score, –0.342) (Fig. 1D). A heatmap visualizing the 10 leading genes of this GSEA showed good clustering over biological replicates and therefore small variability (Fig 1E). Many prominent genes were directly linked to actin cytoskeleton organization, such as *FAT1*,^41^ and were highly downregulated in response to OSM treatment (Fig. 1E).

In summary, our data confirm the proangiogenic effect of OSM on vascular endothelial cells in vitro*.* Furthermore, RNA sequencing data indicated a link between OSM and inflammatory processes, as well as changes in the actin cytoskeleton transduced by STAT3 signaling.

### OSM Limits Neovascularization and Vaso-Obliteration in the OIR Model

**Figure 2.**
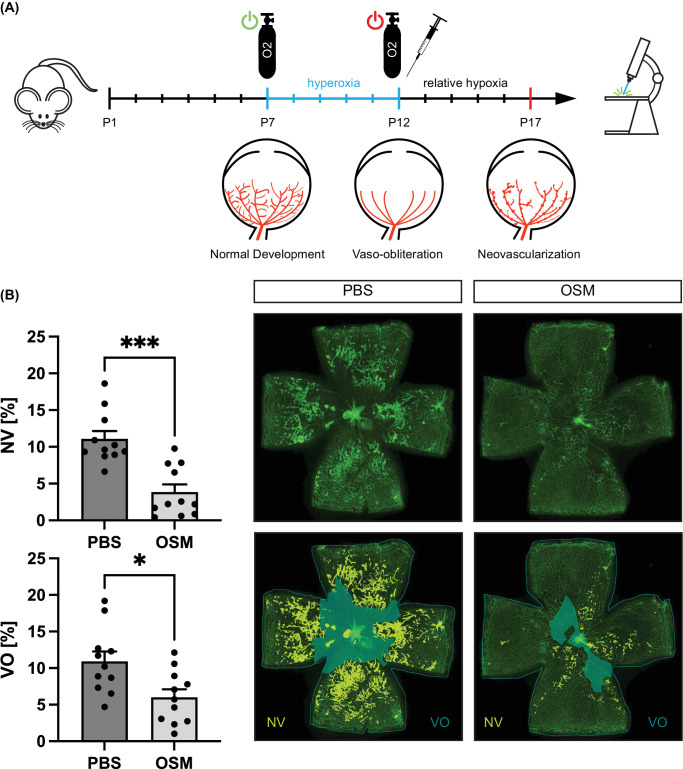
OSM injection reduced the area of neovascularization and vaso-obliteration in the OIR model. (**A**) Graphical visualization of the OIR setup. P7 pups were exposed to 75% oxygen until P12. OSM or PBS control injections were conducted at the end of oxygen treatment. Eyes were collected at P17 and stained for immunohistochemistry. (**B**) Quantification of NV and VO in OIR P17 mice following OSM or PBS control treatment. *N* = 11 mice per condition from three independent OIR experiments.

Based on our observations in vitro, we next wanted to investigate whether the proangiogenic effect of OSM on vascular endothelial cells would translate in vivo in the context of a hypoxia-driven retinal disease model. In the mouse model of OIR, pups received intravitreal injections of 50 pg OSM or PBS as control at OIR P12, the beginning of the hypoxic phase that elicits VEGF-driven angiogenesis (Fig. 2A). OSM significantly reduced the amount of pathological NV present at OIR P17 to 34.8% of the amount measured in PBS-treated controls (Fig. 2B). The area of VO was also significantly reduced by OSM treatment to 54.5% compared to PBS-treated eyes (Fig. 2B). These data suggest that OSM is capable of modifying angiogenesis by limiting development of aberrant vessels and enhancing revascularization in vivo despite its well-known pro-angiogenic effect on vascular endothelial cells in vitro*.*^42^

### OSM Activates Müller Cells

**Figure 3.**
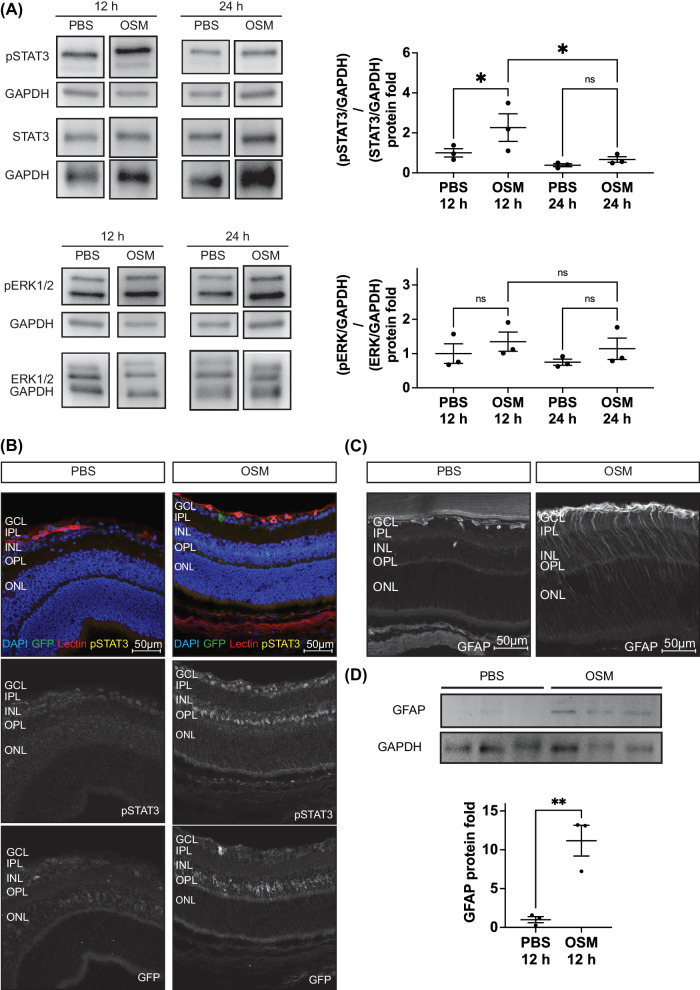
Müller cells were activated in response to OSM treatment. (**A**) Western blot analysis of whole retina lysates for pSTAT3 and pERK 12 and 24 hours after injection of OSM or PBS control. Representative blots are shown. *N* = 3 biological replicates out of three independent OIR experiments. A one-way ANOVA adjusted for multiple testing was used for statistical analysis. (**B**) Representative images of retinal cryosections 12 hours after intravitreal injection with OSM or PBS control at OIR P12 using ALDH1L1-GFP^+^ transgenic mice. The GCL, inner plexiform layer (IPL), inner nuclear layer (INL), outer plexiform layer (OPL), and outer nuclear layer (ONL) are highlighted. (**C**) Representative images of retinal cryosections of non-transgenic mice 12 hours after intravitreal injection with OSM or PBS control at OIR P12. (**D**) Western blot analysis of murine whole retina lysates for GFAP 12 hours after OSM or PBS injection at OIR P12. *N* = 3 biological replicates (three mice). A two-tailed *t*-test was used for statistical analysis.

To elucidate the mechanism behind the angiomodulative effect of OSM in vivo we next aimed to identify general signaling patterns in the retina. For that purpose, retinal samples were collected 12 and 24 hours after injection for western blot analysis to characterize OSM-dependent signaling. Western blot analysis of whole retinal lysates revealed OSM-dependent activation of the STAT3 signaling pathway characterized by increased phosphorylated STAT3 (pSTAT3) levels at 12 hours after injection; however, these levels decreased rapidly 24 hours after injection (Fig. 3A). Analysis for OSM-induced activation of ERK represented by phosphorylated ERK (pERK) did not support significant activation at 12 hours or 24 hours after injection (Fig. 3A). Considering the early and pronounced increase in pSTAT3 signaling, we next employed immunohistochemistry on retinal cross-sections 12 hours after OSM treatment to localize pSTAT3. Using transgenic mice that express green fluorescent protein (GFP) linked to ALDH1L1, a Müller glia cell marker,^43^ we identified prominent pSTAT3 signal in the inner nuclear layer (INL), where nuclei of Müller cells are located, as well as in the ganglion cell layer (GCL), were ganglion cell nuclei, and isolectin positive vascular structures (Fig. 3B). The pSTAT3 signal in the INL aligned with the GFP signal of the ALDH1L1-GFP^+^ transgenic mice, which was also vastly enhanced after OSM injection compared to PBS-injected mice, suggesting upregulation of ALDH1L1 due to treatment. The enhanced pSTAT3 signal in the INL was reproduced in two different, non-transgenic mice (Supplementary Fig. S2A). Immunohistochemistry of non-transgenic mice revealed further enhanced glial fibrillar acidic protein (GFAP) due to OSM injection spanning from the INL to the GCL, which correlates with the typical spatial distribution of GFAP expression in activated Müller cells (Fig. 3C).^44^^,^^45^ We reproduced the staining with two different mice (Supplementary Fig. S2B) and further confirmed significantly elevated GFAP levels due to OSM by western blot in whole retina cell lysate 12 hours after OSM injection (Fig. 3D). In total, these data highlight the responsiveness of Müller cells in the OIR model to OSM injection.

### In Vitro, Müller Cells Respond to OSM Treatment With Changes in Their Secretome

**Figure 4.**
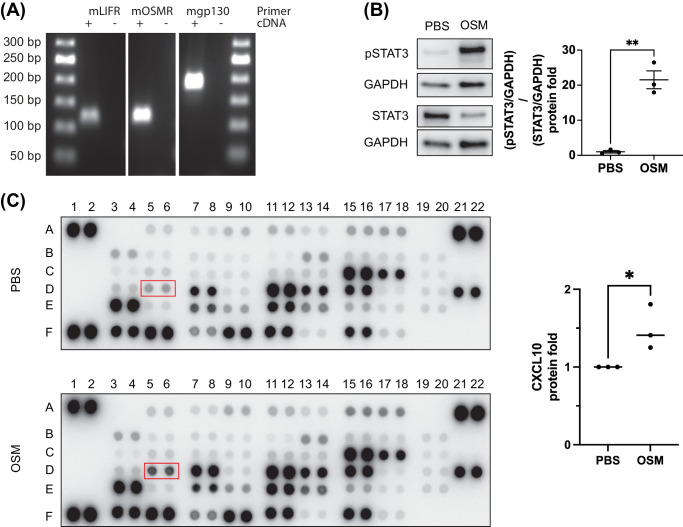
Müller glia cells responded to OSM stimulation in vitro via STAT3 activation. (**A**) RT-PCR analysis of RNA from primary Müller cells. (**B**) Western blot analysis of Müller cell lysates for pSTAT3 and STAT3 15 minutes after stimulation with OSM or PBS control. Representative images are shown; statistical analysis was performed on three independent biological replicates. A two-tailed *t*-test was used for statistical analysis. (**C**) Analysis of secreted protein in Müller cell conditioned media 72 hours after treatment with OSM or PBS for 15 minutes using the Proteome Profiler Mouse Angiogenesis Array Kit. Representative images are shown; statistical analysis for CxCl10 was performed on three independent biological replicates. A two-tailed *t*-test was used for statistical analysis.

To better understand how Müller cells process OSM signaling, a primary Müller cell culture model was established. RT-PCR analysis confirmed that murine primary Müller cells express the receptor components gp130, LIFR, and OSMR necessary for OSM-induced signaling (Fig. 4A). Stimulating primary Müller cells in vitro with OSM resulted in a clear pSTAT3 signal compared to controls (Fig. 4B), analogous to the in vivo situation. As secreted cytokines might be important mediators in Müller glia and vascular endothelial cell interactions, we next analyzed the supernatant of primary Müller cells after short stimulation with OSM or PBS control for changes in the cytokine composition using a proteome profiler (Fig. 4C). Interestingly, the antiangiogenic cytokine CXCL10^14^ was significantly increased in the supernatant of OSM-stimulated Müller cells (Fig. 4C). Taken together, these data indicate that Müller cells in vitro and in vivo are highly responsive to OSM activating the STAT3 signaling pathway, which in turn alters their secretome.

### Retinal Vascular Endothelial Cells Can Be Enriched From OIR Retinas Using Fluorescence-Activated Cell Sorting

**Figure 5.**
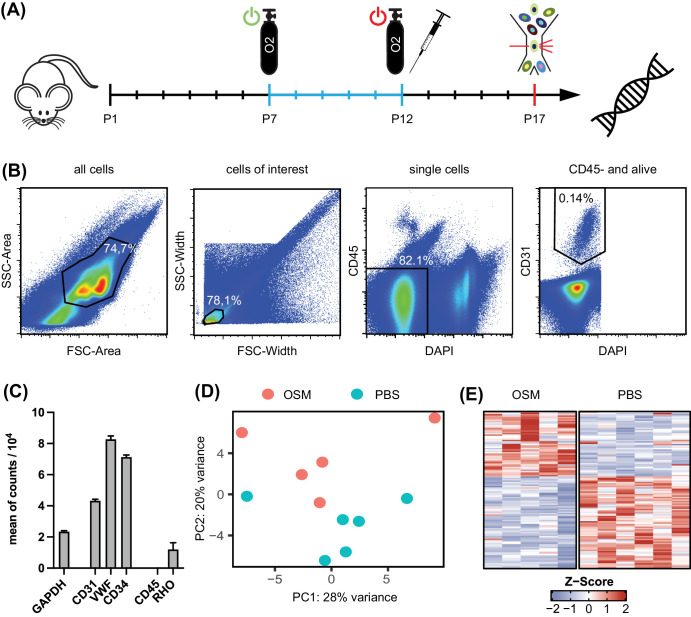
Retinal vascular endothelial cells from mice at OIR P17 can be enriched by FACS and subsequently employed for RNA sequencing. (**A**) Graphical visualization of the experimental setup to isolate retinal vascular endothelial cells from OIR mice following OSM treatment to generate samples of sorted vascular endothelial cells for RNA sequencing analysis. At OIR P17, retina samples were digested, stained, and sorted for CD34^+^/CD45^−^ vascular endothelial cells. (**B)** Representative gating strategy for sorting retinal vascular endothelial cells. (**C**) Bar graphs visualizing the enriched expression of specific vascular endothelial cell marker according to the sequencing data. For reference, GAPDH and CD45 are also shown. Data are representative of all 11 samples. (**D**) PCA of vascular endothelial cell samples visualizing the variance in their whole transcriptome. In total, six PBS-treated samples and five OSM-treated samples were analyzed, originating from three independent OIR experiments. (**E**) Heatmap of all 116 DEGs with >25 reads on average using the *Z*-score.

We next aimed to characterize the changes in the transcriptomic profiles of retinal vascular endothelial cells as a result of OSM treatment. Because immunhistochemical stainings for pSTAT3 (Fig. 3B) revealed that multiple retinal cell types, including Müller cells and vascular endothelial cells, respond to OSM early on, we chose OIR P17, the time point of maximum NV, to capture the overall OSM-induced changes in vascular endothelial cells resulting from direct and indirect Müller cell–mediated effects on vascular endothelial cells. At OIR P17, retinal vascular endothelial cells were isolated by fluorescence-activated cell sorting (FACS) for RNA sequencing (Fig. 5A). On average, 2411 vascular endothelial cells could be isolated from two pooled retinas applying the following gating strategy: FSC-A/SSC-A roughly excluded debris and lager clumps. Gating on single cells by FSC-W/SSC-W, as well as excluding dead cells based on a negative DAPI stain and CD45^+^ cells, resulted in 82.1% of all events. From this, 0.14% of cells were CD31^+^ and therefore defined and isolated as a retinal vascular endothelial cell population (Fig. 5B). Transcriptomic analysis of these samples showed enrichment of specific vascular endothelial cell markers, including CD31, CD34, and von Willebrand factor (VWF) in comparison to housekeeping genes (e.g., *GAPDH*) and photoreceptor markers (e.g., *rhodopsin*) (Fig. 5C), giving us confidence in our isolation protocol for vascular endothelial cells. Interestingly, the data revealed that the sorted cells indeed expressed gp130, OSMR, and LIFR, but not CNTFR (Supplementary Fig. S3).

### RNA Sequencing Revealed Longlasting Anti-Angiogenic and Antiproliferative Changes in the Expression Profile of Vascular Endothelial Cells 5 Days After OSM Treatment

PCA was used to visualize the variance between OSM-treated samples at P12 and control groups, as well as biological replicates. Despite considerable variance, a difference was seen as represented by the second principal component (PC2) (Fig. 5D). We identified 173 DEGs, and 123 DEGs had an absolute log_2_ fold change > 1. We found that 74 genes were upregulated in the OSM-injected samples, and 99 were downregulated. All DEGs with at least 25 counts were visualized in a heatmap (Fig. 5E); all DEGs with their respective log_2_ fold changes and adjusted *P* values can be found in Supplementary File S2.

**Figure 6.**
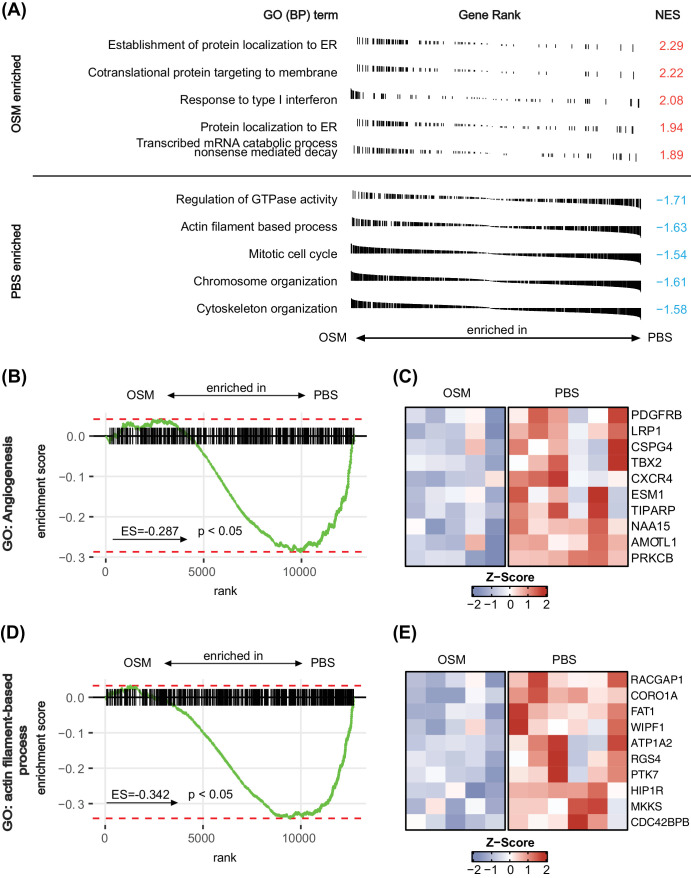
Detailed analysis of the transcriptomic differences in retinal vascular endothelial cells of OSM- versus PBS-treated eyes. (**A**) Summary of GSEA results of the five most enriched biological processes according to GO terms sorted by their adjusted *P* values. A positive NES indicates enrichment in the OSM-treated group, whereas negative scores indicate enrichment in PBS-treated samples. (**B**) GSEA for the GO term “angiogenesis” (GO:0001525) using shrunken log_2_ fold changes for ordering genes. A positive enrichment score indicates enrichment in OSM-treated samples. (**C**) Heatmap of 10 leading-edge genes of the GSEA from the GO term “angiogenesis” (GO:0001525) visualizing their expression in OSM-treated samples in comparison to PBS-treated samples using the *Z*-score. (**D**) GSEA for the GO term “actin filament-based process” (GO:0030029) using shrunken log_2_ fold changes for ordering genes. A positive enrichment score indicates enrichment in OSM samples. (**E**) Heatmap of 10 leading-edge genes of the GSEA from the GO term “actin filament-based process” (GO:0030029) visualizing their expression in OSM-treated samples in comparison to PBS-treated samples using the *Z*-score.

To gain an overview over the biological processes in vascular endothelial cells that are mainly affected by OSM injection, we used GSEA to screen through all gene sets of biological processes of the GO term database. Looking at the five most significantly enriched or depleted gene sets according to the adjusted *P* values in GSEA, we detected an enriched “response of type 1 interferon” (GO:0034340) with a normalized enrichment score (NES) of 2.08 in the OSM group, a score that is often associated with antiangiogenic effects,^46^ and a depletion of genes related to the activity of the “mitotic cell cycle” (GO:0000278), which corresponds to reduced endothelial cell proliferation in OSM-treated samples (Fig. 6A). Also, many GO terms required for mitosis and therefore cell proliferation such as “chromosome organization” (GO:0051276) or “actin filament-based process” (GO:0030029) were also depleted in OSM-treated samples (Fig 6A). All details, including the leading-edge genes, can be found in Supplementary Tables S8 and S9.

Because angiogenesis-associated genes play an important role in retinal vasoproliferative disease, we specifically checked for the GO term :angiogenesis: (GO:0001525) in GSEA and saw significant enrichment represented by an enrichment factor of –0.287 (Fig. 6B). The heatmap visualizes leading-edge genes known for their impact on angiogenesis, including *PDGFRB* or *LRP1*, which were downregulated in response to OSM injection 5 days before at P12 (Fig. 6C).

As the gene set of “actin filament-based process” (GO:0030029) showed a strong depletion in our in vivo and in vitro datasets, we wanted to take a more detailed look at the genes contributing to this depletion in OSM-injected eyes (Fig. 6D). Again, the top 10 genes in this GSEA presented downregulation of prominent members responsible for the control of the actin cytoskeleton in OSM samples such as FAT1, which was also a key member in the in vitro analysis (Fig. 6E). Because one prominent reason for the development of neovascularization in the OIR model is the dysregulated formation of tip and stalk endothelial cells,^47^ we further investigated whether any changes in the Notch pathway, a major regulator of tip/stalk cell differentiation, might be present 5 days after OSM treatment.^47^ Again using GSEA, we identified no significant enrichment (Supplementary Fig. S4A), whereas the expression of major genes involved in the regulation of correct tip and stalk cell formation, such as *NOTCH1*, *JAG1*, and *DLL*,^47^^,^^48^ was also not changed (Supplementary Fig. S4B).

**Figure 7.**
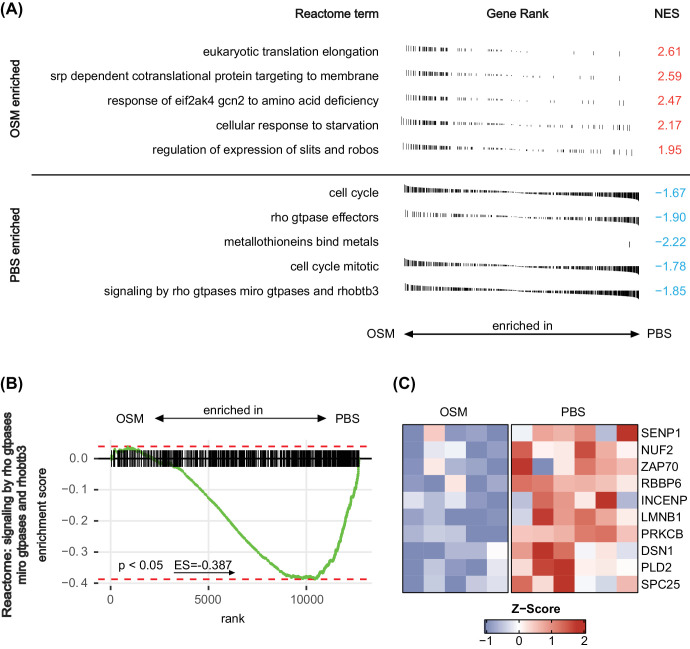
Reactome analysis of the sequencing data from retinal vascular endothelial cells of OSM- versus PBS-treated eyes. (**A**) Summary of GSEA results of the five most enriched Reactome terms sorted by their adjusted *P* values. A positive NES indicates enrichment in the OSM-treated group, whereas negative scores indicate enrichment in PBS-treated samples. (**B**) GSEA for the Reactome term “signaling by Rho GTPases, Miro GTPases and RHOBTB3” (R-HSA-9716542) using shrunken log_2_ fold changes for ordering genes. A positive enrichment score indicates enrichment in OSM samples. (**C**) Heatmap of 10 leading-edge genes of the GSEA from the Reactome term “signaling by Rho GTPases, Miro GTPases and RHOBTB3” visualizing their expression in OSM-treated samples in comparison to PBS-treated samples using the *Z*-score.

We then aimed to expand the RNA sequencing analysis to include molecular processes using the Reactome database for GSEA. A closer look at the five most enriched and depleted pathways according to the adjusted *P* values revealed that influence on the mitotic cell cycle was predominantly represented by depletion of cell cycle-associated Reactome Terms, as for example “cell cycle” (R-HSA-1640170), by a NES of –1.67 (Fig. 7A). Looking closer at this Reactome pathway (Fig. 7B) and its leading-edge genes, we found that *PRKCB* was one of the 10 most influential genes. Another gene among the top 10 leading-edge genes was *PLD2*, which is a prominent guanine nucleotide exchange factor^49^ (Fig. 7C). All details, including all leading-edge genes, can be found in Supplementary Tables S10 and S11.

In summary, the RNA sequencing data obtained from sorted retinal vascular endothelial cells following OSM treatment at P12 affirm the capacity of OSM to induce lasting changes in the angiogenic activity of endothelial cells.

## Discussion

For cytokines of the IL-6 family, divergent and even contradictory angiomodulatory effects have been reported.^17^ In the case of OSM, we and other researchers have observed pro-angiogenic effects on vascular endothelial cells in vitro*.*^17^^,^^42^ RNA sequencing analysis in the in vitro part of this study underlined the relevance of STAT3 signaling and STAT3 targets (e.g., SOCS3) to the signaling process. The in vivo part of this project revealed that OSM can exert different nuanced regulatory effects in more complex multicellular systems such as the retina. Although OSM acts as a strong pro-angiogenic agent on vascular endothelial cells in vitro, its angiomodulatory effect in the retina seems to be determined by activating Müller cells to reduce NV and VO.

Interestingly, earlier studies on CNTF yielded comparable results for OSM in in vivo angiogenesis models,^13^ including activation of the STAT3 signaling pathway in vascular endothelial cells and Müller cells, which correlated with a strong reduction of retinal neovascularization.^14^ In vitro*,* CNTF and OSM induce similar changes in the Müller cell secretome, including upregulation of CXCL10. We previously established CXCL10 as an antiangiogenic agent both in vitro and in vivo in different mouse models of vasoproliferative eye diseases as the VLDR or laser choroidal neovascularization (CNV) model.^14^ Beyond the eye, CXCL10 is well known for reducing angiogenesis in sarcoma,^50^ after spinal cord injury,^51^ or in the heart,^52^ suggesting that CXCL10 could be a noteworthy candidate for further research concerning vasoproliferative eye diseases.

In the OIR model, OSM was able to reduce not only NV but also VO. This observed pattern aligns with findings from our earlier investigations involving CNTF^13^ and appears to be a frequent phenomenon in the OIR model. For example, the injection of IL-12,^53^ application of OTX008 as a small molecular inhibitor,^54^ or utilization of the licensed anti-VEGF treatment option Conbercept^55^ resulted in the same changes in the OIR phenotype. The molecular processes underlying this angiomodulatory phenotype remain inadequately understood, and the question persists whether it is a phenomenon specific to the OIR model or the manifestation of a more intricate mechanism inherent to these agents. As Müller cell activation was observed in response to both IL-6 family cytokines CNTF and OSM, these cells might play a central role in mediating vascular changes. Müller cells are known to be a major source of pro- as well as anti-angiogenic cytokines,^56^ including pigment epithelium-derived factor (PEDF)^57^ or CXCL10.^14^ Furthermore, direct cell–cell interactions through connexins and gap junctions represent another way of communication between Müller cells and retinal vascular endothelial cells.^58^^,^^59^ Further experiments including single-cell RNA sequencing or conditional knock-out mouse models could shed more light on the complex effect of OSM on the interplay of Müller cells and vascular endothelial cells in the context of the OIR model. Despite significant limitations of in vitro settings, particularly due to cultivation-induced changes in Müller cells,^60^ co-culture experiments may represent a different approach to study OSM-driven molecular interactions of Müller cells and vascular endothelial cells in more detail.

Our immunohistochemical staining with prominent pSTAT3 labeling in the GCL as well as INL suggests that multiple cell types respond to OSM following intravitreal injection. Among those, Müller cells could be identified as an important cell population shaping the effect of OSM on the retina. In the INL, nuclei of amacrine cells, horizontal cells, or macrophages/microglia are also localized. The GCL harbors ganglion cells and vascular endothelial cells. The impact of those cell types on the angiomodulatory effect of OSM currently remains elusive. Single-cell RNA sequencing would help to better understand and showcase their role. However, small cell populations such as retinal vascular endothelial cells are often insufficiently represented in these analyses. We do believe that characterizing transcriptomic changes in vascular endothelial cells as the effector cells for retinal vasoproliferative disease represents a valuable first approach to unraveling differences in the effects of OSM in vitro and in vivo.

One significant distinction between in vitro and in vivo settings for investigating angiogenesis lies in the more intricate hierarchy of tip and stalk cells formed by vascular endothelial cells in vivo*.*^48^ According to recent literature, a primary cause for the formation of NV is the dysregulated development of these hierarchies due to Notch signaling.^47^ In our RNA sequencing data OSM injection at P12 did not affect any signaling patterns related to tip/stalk cell formation at P17. However, in a bulk RNA sequencing approach that includes all vascular endothelial cells, the background noise might be too high to distinguish these effects. Furthermore, sequencing at an earlier time point, such as P13, could also yield distinct data, as our observations at P17 primarily capture the long-term alterations induced by OSM treatment, contrasting with the acute shifts immediately after treatment.

Regarding the interpretation of our study results, certain limitations should be considered: The sequencing data indicate a significant level of variability between individual samples (Fig. 5D). This may be attributed to the relatively small cell number of retinal vascular endothelial cells isolated from OIR retinas as well as other confounding parameters such as variability of intravitreal injections, independent OIR experiments,^61^^,^^62^ and the genetic diversity of mice. Furthermore, the time point of sampling 5 days after treatment may represent another source of variability. Earlier time points such as OIR P14 might have revealed stronger transcriptomic shifts due to a shorter interval to injection. However, OIR P17 was chosen deliberately as it represents the time point of maximum NV in the OIR model,^63^ implicating a culmination of the effect of OSM on vascular endothelial cells. Furthermore, comparison of STAT3 activation patterns in vitro^17^ and in vivo indicated a delayed STAT3 activation pattern in vivo with significant activation at 12 hours after injection, whereas the pSTAT3 signal declined in vitro after 1 hour. Later time points for RNA sequencing analysis for in vivo samples thus appeared to be more appropriate. To better understand the complex effects of OSM on vascular endothelial cells in vivo in comparison to in vitro, a time-course experiment at P13, P14, P17, and P21 would help to distinguish early most likely direct effects on vascular endothelial cells from long-lasting effects that integrate changes induced by other cell types at P17. The observed enrichment of pathways and gene shifts measured in this study should therefore be interpreted not as the acute impact of OSM but rather as its long-term influence on the pathophysiological process of NV formation. Unfortunately, in vivo time-course experiments are challenging due to high RNA sequencing costs, whereas low RNA yields from sorted retinal cell types limit standard qPCR analysis.

In summary, this study indicates that angiomodulatory agents such as OSM may have unexpected and sometimes contradictory effects in vivo compared to in vitro depending on the cell types affected in a complex multicellular system such as the retina. OSM has a beneficial angiomodulatory effect on retinal angiogenesis, with Müller cells serving as essential elements in vascular homeostasis.

## Supplementary Material

Supplement 1

Supplement 2

Supplement 3

Supplement 4
