# Supplementary material for: Oncostatin M Reduces Pathological Neovascularization in the Retina Through Müller Cell Activation
Source: Invest Ophthalmol Vis Sci. 2024 Jan 8;65(1):22. doi: 10.1167/iovs.65.1.22 (PMC10777876; doi:10.1167/iovs.65.1.22)

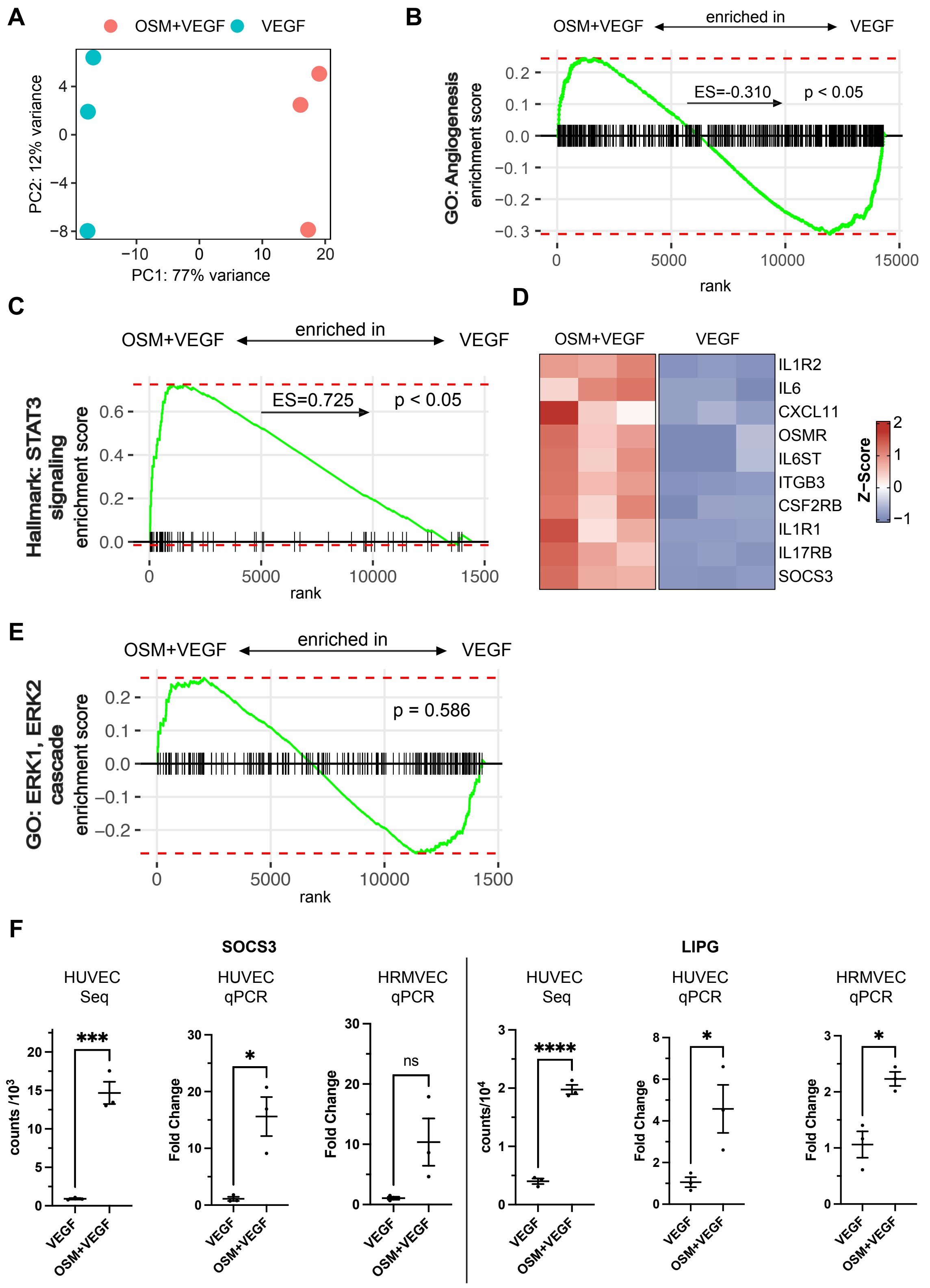

Supplemental Figure S1: Supplemental data of the *in vitro* RNA sequencing. (A) Principal component analysis of the RNA sequencing data between OSM+VEGF and

VEGF stimulated HUVECs. (**B**) GSEA of RNA sequencing data for the GO term "Angiogenesis" (GO:0001525) between OSM+VEGF and VEGF stimulated HUVECs. A positive enrichment score refers to enrichment of the specific set for OSM+VEGF incubated HUVECs in contrast to cells just stimulated by VEGF. (**C**) GSEA of RNA sequencing data for the Hallmark "IL6 Jak Stat3 signaling" gene set between OSM+VEGF and VEGF stimulated HUVECs. A positive enrichment score refers to enrichment of the specific set for OSM+VEGF incubated HUVECs in contrast to cells just stimulated by VEGF. (**D**) Heatmap of 10 leading edge genes of the GSEA Hallmark "IL6 Jak Stat3 signaling" gene set visualizing their expression in OSM+VEGF treated samples in comparison to VEGF samples using the Z-score. (**E**) GSEA of RNA sequencing data for the GO term "ERK1 and ERK2 cascade" (GO:0070371) between OSM+VEGF and VEGF stimulated HUVECs. A positive enrichment score refers to enrichment of the specific set for OSM+VEGF incubated HUVECs in contrast to cells just stimulated by VEGF. (**F**) Validation of selected RNA sequencing results by qPCR of HUVECs or HRMVECs stimulated for 24 h with VEGF or OSM+VEGF. Data represents samples from three independent experiments.

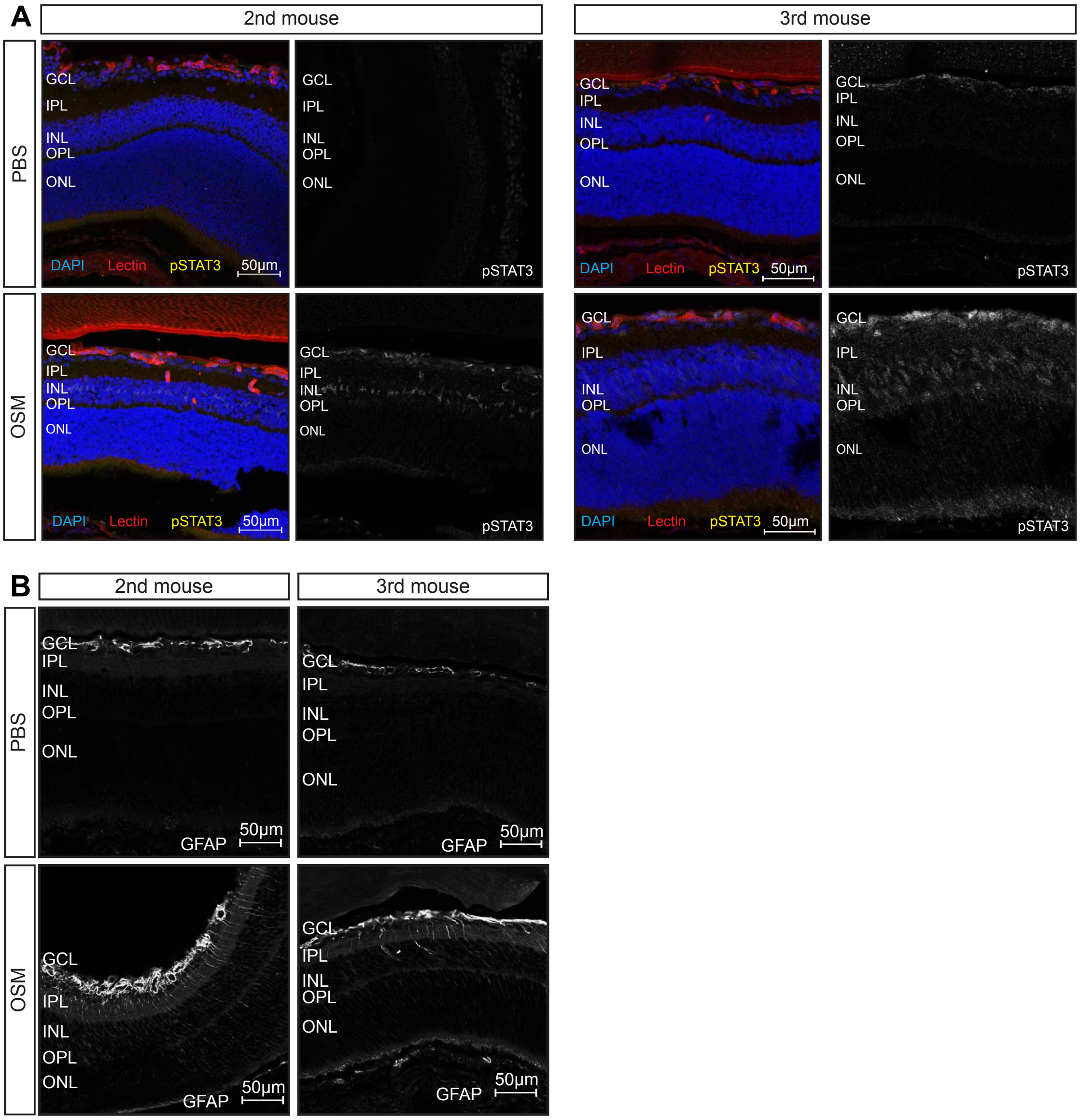

Supplemental Figure S2: Müller cells respond to OSM *in vivo*. (**A**) Representative images of retinal cryosections from non-transgenic mouse 2 and mouse 3 12 h after intravitreal injection with OSM or PBS control at OIR P12 stained for DAPI, lectin and pSTAT3. (**B**) Representative images of retinal cryosections from non-transgenic mouse 2 and mouse 3 12 h after intravitreal injection with OSM or PBS control at OIR P12.

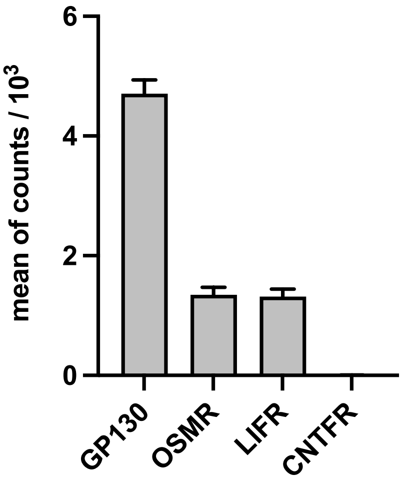

Supplemental Figure S3: Sorted retinal vascular endothelial cells express all components of the OSM receptor complex. Bar graphs visualizing the expression of gp130, OSMR, LIFR and CNTFR according to the sequencing data. Data are representative of all 11 samples.

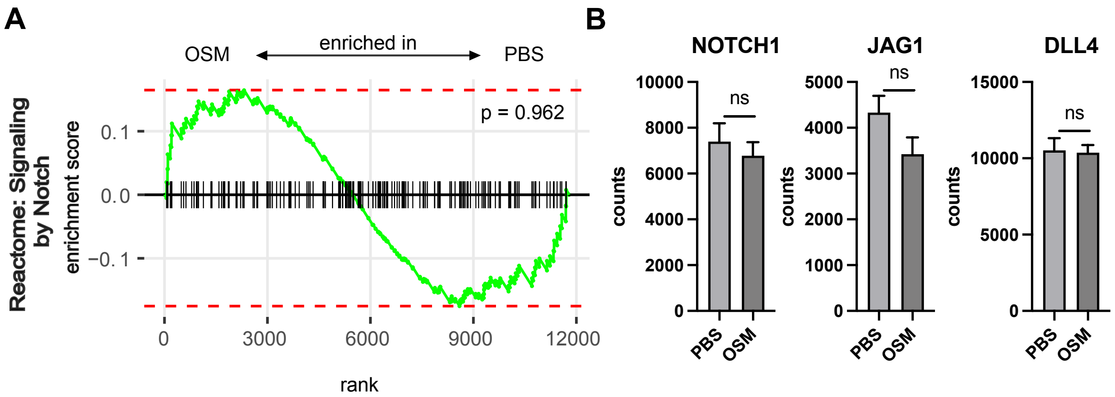

Supplemental Figure S4: Notch signaling is not altered by OSM injection in sorted retinal vascular endothelial cells. (**A**) GSEA for the Reactome pathway ‘Signaling by NOTCH’ (R-GGA-157118) using shrunken log2-fold changes for ordering genes. Positive enrichment score refers to enrichment in OSM-treated eyes. (**B**) Bar graphs visualizing the expression of different NOTCH related genes according

to their counts in the RNA sequencing data set of sorted retinal vascular endothelial cells from the OIR model with or without intravitreal OSM injection at P12.

Uncut Western Blots

Supplemental Figure S5:

(I) pSTAT3 and pERK for mice from OIR 12 h or 24 h post P12 with or without OSM injection

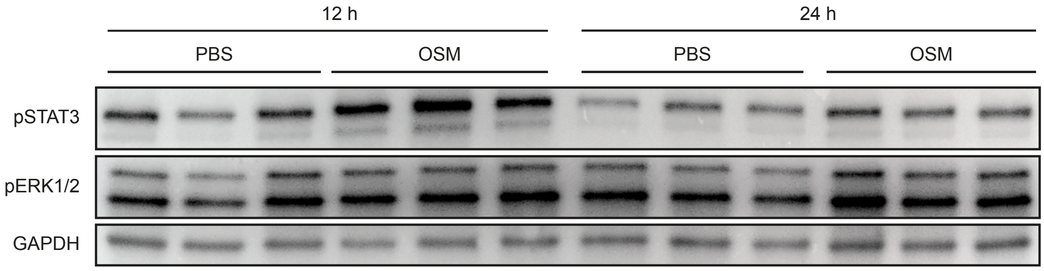

(II) STAT3 for mice from OIR 12 h or 24 h post P12 with or without OSM injection

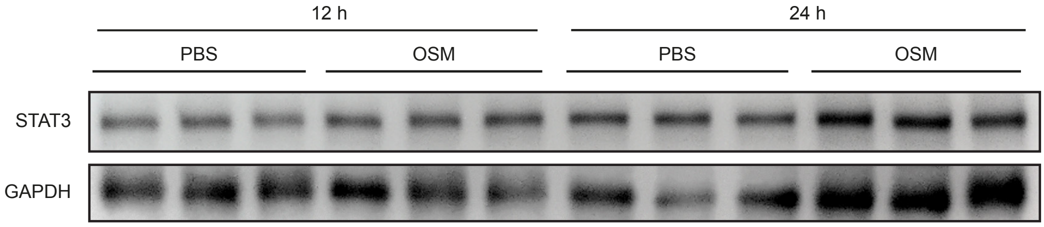

(III) ERK for mice from OIR 12 h or 24 h post P12 with or without OSM injection

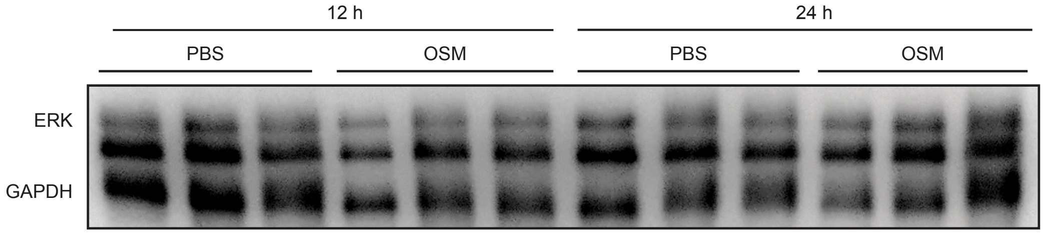

(IV) pSTAT3 for Müller cells with or without OSM stimulation

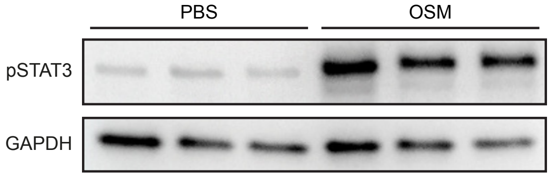

(V) STAT3 for Müller cells with or without OSM stimulation

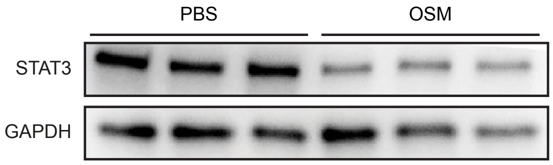

(**VI**) Angiogenesis Proteome Profiler n=2-3

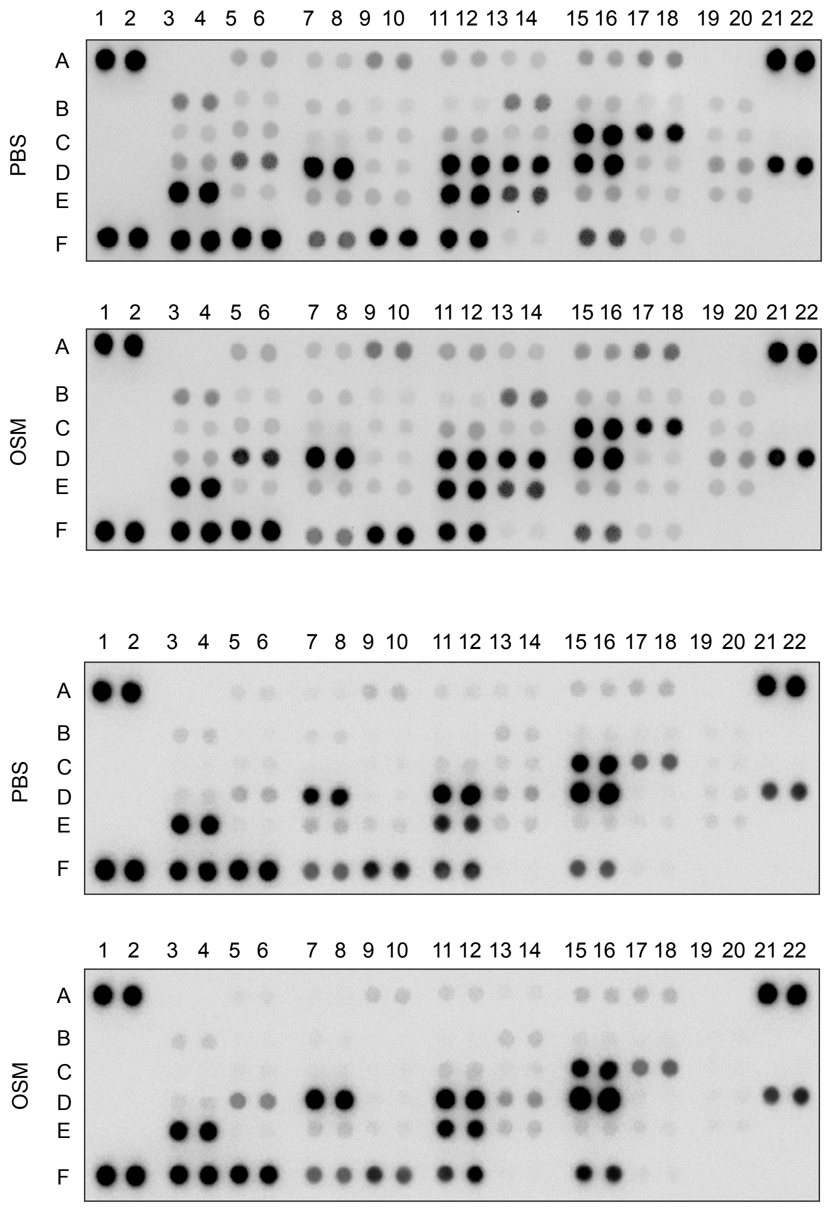

Supplement: Supplement 1 [file iovs-65-1-22_s001.pdf]
