# Supplementary material for: Oncostatin M Reduces Pathological Neovascularization in the Retina Through Müller Cell Activation
Source: Invest Ophthalmol Vis Sci. 2024 Jan 8;65(1):22. doi: 10.1167/iovs.65.1.22 (PMC10777876; doi:10.1167/iovs.65.1.22)
Supplement: Supplement 2 [file iovs-65-1-22_s002.pdf]

**Supplemental Table 1: Targets of the Proteome Profiler Angiogenesis Array kit and their coordinates.**

| **Coordinate** | **Target** |
|---|---|
| A1, A2 | Reference Spots |
| A5, A6 | ADAMTS1 |
| A7, A8 | Amphiregulin |
| A9, A10 | Angiogenin |
| A11, A12 | Ang-1 |
| A13, A14 | Ang-3 |
| A15, A16 | Coagulation Factor III |
| A17, A18 | CXCL16 |
| A21, A22 | Reference Spots |
| B3, B4 | Cyr61 |
| B5, B6 | DLL4 |
| B7, B8 | DPPIV |
| B9, B10 | EGF |
| B11, B12 | Endoglin |
| B13, B14 | Endostatin/Collagen XVIII |
| B15, B16 | Endothelin-1 |
| B17, B18 | FGF acidic |
| B19, B20 | FGF basic |
| C3, C4 | KGF |
| C5, C6 | Fractalkine |
| C7, C8 | GM-CSF |
| C9, C10 | HB-EGF |
| C11, C12 | HGF |
| C13, C14 | IGFBP-1 |
| C15, C16 | IGFBP-2 |
| C17, C18 | IGFBP-3 |
| C19, C20 | IL-1α |
| C21, C22 | IL-1β |
| D3, D4 | IL-10 |
| D5, D6 | CXCL10 |
| D7, D8 | KC |
| D9, D10 | Leptin |
| D11, D12 | MCP-1 |
| D13, D14 | MIP-1α |
| D15, D16 | MMP-3 (pro and mature form) |
| D17, D18 | MMP-8 (pro form) |
| D19, D20 | MMP-9 (pro and active form) |
| D21, D22 | NOV |
| E3, E4 | Osteopontin |
| E5, E6 | PD-ECGF |
| E7, E8 | PDGF-AA |
| E9, E10 | PDGF-AB/PDGF-BB |
| E11, E12 | Pentraxin-3 |
| E13, E14 | Platelet Factor 4 |

| | |
|---|---|
| E15, E16 | PlGF-2 |
| E17, E18 | Prolactin |
| E19, E20 | Proliferin |
| F1, F2 | Reference Spots |
| F3, F4 | SDF-1 |
| F5, F6 | Serpin E1 |
| F7, F8 | Serpin F1 |
| F9, F10 | Thrombospondin-2 |
| F11, F12 | TIMP-1 |
| F13, F14 | TIMP-4 |
| F15, F16 | VEGF |
| F17, F18 | VEGF-B |
| F19, F20 | Negative Control |

**Supplemental Table 2: Antibodies and their dilutions.**

| antigen | Cat# | supplier | dilution |
|---:|---:|---:|---:|
| pSTAT3 | 9145 | Cell Signaling | 1:100 (IHC), 1:2000 (WB) |
| STAT3 | 4904 | Cell Signaling | 1:2000 (WB) |
| pERK | 4370 | Cell Signaling | 1:2000 (WB) |
| ERK | 4695T | Cell Signaling | 1:1000 (WB) |
| GAPDH | MAB374 | Merck | 1:10000 (WB) |
| GFAP | ab4674 | Abcam | 1:1000 (IHC), 1:1000 (WB) |
| CD16/CD32 | 553131 | BD | 1:200 (FC) |
| CD31 | 12-0211-82 | Thermo Fisher | 1:100 (FC) |
| CD45 | 56-0451-82 | Thermo Fisher | 1:100 (FC) |

**Supplemental Table 3: Primers used for RT-PCR and qPCR**

| target | forward | reverse | product size |
|---:|---:|---:|---:|
| mgp130 | CCGTGTGGTTACATCTACCCT | CGTGGTTCTGTTGATGACAGTG | 180 |
| mOSMR | CATCCCGAAGCGAAGTCTTGG | GGCTGGGACAGTCCATTCTAAA | 110 |
| mLIFR | TACGTCGGCAGACTCGATATT | TGGGCGTATCTCTCTCTCCTT | 113 |
| hSOCS3 | CCTGCGCCTCAAGACCTTC | GTCACTGCGCTCCAGTAGAA | 99 |
| hLIPG | GATGGACGATGAGCGGTATCT | CGCATCCGTGTAAAGCTGG | 131 |

**Supplemental Table 4: RNA quality and sequencing depth of the *in vitro* RNA sequencing of HUVECs.**

| sample | RIN | targeted depth | actual depth |
|---:|---:|---:|---:|

| | | | |
|---|---|---|---|
| VEGF_1 | 9.9 | 30,000,000 | 40,215,996 |
| VEGF_2 | 9.9 | 30,000,000 | 39,871,360 |
| VEGF_3 | 9.8 | 30,000,000 | 41,586,581 |
| OSM+VEGF_1 | 9.8 | 30,000,000 | 43,014,719 |
| OSM+VEGF_2 | 9.9 | 30,000,000 | 44,925,481 |
| OSM+VEGF_3 | 9.9 | 30,000,000 | 43,204,360 |

**Supplemental Table 5: RNA quality and sequencing depth of the *in vivo* RNA sequencing of sorted murine retinal endothelial cells.**

| sample | RIN | targeted depth | actual depth |
|---:|---:|---:|---:|
| PBS_1 | 9 | 40,000,000 | 45,880,364 |
| PBS_2 | 8.2 | 40,000,000 | 43,856,670 |
| PBS_3 | 8.5 | 40,000,000 | 48,700,954 |
| PBS_4 | 7.8 | 40,000,000 | 50,130,437 |
| PBS_5 | 8 | 40,000,000 | 47,291,514 |
| PBS_6 | 7.3 | 40,000,000 | 44,003,163 |
| OSM_1 | 8.7 | 40,000,000 | 48,553,465 |
| OSM_2 | 8.7 | 40,000,000 | 48,057,344 |
| OSM_3 | 7.2 | 40,000,000 | 42,999,986 |
| OSM_4 | 7.4 | 40,000,000 | 40,489,298 |
| OSM_5 | 10 | 40,000,000 | 81,321,961 |

**Supplemental Table 6: Detailed results of the top five enriched GO terms (biological processes) obtained from the GO enrichment analysis conducted on the *in vitro* RNA sequencing data. Enrichment refers to upregulation in OSM+VEGF vs VEGF controls.**

| ID | Description | Count | GeneRatio | padj | geneID |
|---:|---|---:|---:|---:|---:|
| GO:0006954 | inflammatory response | 88 | 88/863 | 1.15E-09 | SERPINF1/HGF/ACKR1/CIITA/NPPA/IL1R2/MS4A2/PGLYRP1/IL17RB/SPP1/NLRC4/TAC4/CXCR6/GPR17/CCL16/NOX1/IDO1/CCL23/ACP5/CXCL2/C2CD4A/IL1R1/SOCS3/IL1RL2/SELE/IL33/GPR68/IL23A/IL6/MMP25/FOS/CXCL5/TNIP3/TLR5/AFAP1L2/IL15/BCL6/CXCL11/IL31RA/BDKRB2/NAMPT/SCN9A/SELP/OSMR/SEMA7A/CXCL3/CYP26B1/PLSCR1/SBNO2/TIMP1/C2CD4B/NFKBIZ/IL18R1/PRKCQ/IL6ST/CALCRL/LY96/CASP1/IL17RA/IL6 |

| | | | | | |
|---|---|---|---|---|---|
| | | | | | R/CCL2/TNFRSF1A/CHST2/CEBPB/NEAT1/HMOX1/IFI16/ADORA2B/REL/CD47/CYBA/FNDC4/PIK3CG/GJA1/MGST2/NMI/PXK/GSDMD/NFKBIA/STAT3/TGM2/IFNGR1/FOXP1/APOL2/STAT5B/TEK/ITGA2/SIGIRR |
| | | | | | |
| GO:0034097 | response to cytokine | 115 | 115/863 | 3.04E-08 | DUOX1/HGF/CARD14/ACKR1/CIITA/IL1R2/SMPD3/DCST1/CLDN18/IL17RB/CD27/CXCR6/GPR17/CCL16/RHOU/MSC/OPRD1/CCL23/ACP5/CXCL2/IL1R1/SOCS3/IL1RL2/SELE/IL33/CEBPD/IL7/RIPOR2/AVPR2/IL23A/IL6/IFITM1/JAK3/FOS/PTPN13/CXCL5/CSF3/IL15/BCL6/MRC1/CXCL11/IL31RA/OSMR/CSF2RB/IFITM3/LAMP3/ZFP36/SNX10/IFITM2/SOD2/EGR1/CXCL3/ACKR3/GBP2/PARP9/NR5A2/LEPR/PIK3R1/PLSCR1/SBNO2/TIMP1/CEACAM1/IL18R1/IL6ST/RAB20/CXCL16/CASP1/HK2/JUNB/PSMB9/CAMK2D/IL17RA/IL6R/CCL2/CDC42EP4/TNFRSF1A/MAP3K8/CEBPB/HMOX1/IFI16/IFI35/PTP4A3/IL18BP/IL15RA/IFIH1/REL/YBX3/ADAM23/CD47/CYBA/ARID5B/FAS/TRAF3IP2/JAK1/ROBO1/FOXO1/MAP3K5/STAT5A/MAPK11/PSMB8/SLC26A6/NMI/EPS8/RNF138/NLRC5/NFKBIA/STAT3/NFIL3/IFNGR1/STAT5B/UGCG/LTBR/WDR35/SIGIRR/YTHDC2 |
| GO:0071345 | cellular response to cytokine stimulus | 106 | 106/863 | 2.65E-07 | DUOX1/HGF/CARD14/ACKR1/CIITA/IL1R2/SMPD3/DCST1/CLDN18/IL17RB/CD27/CXCR6/GPR17/CCL16/RHOU/MSC/OPRD1/CCL23/CXCL2/IL1R1/SOCS3/IL1RL2/IL33/CEBPD/IL7/RIPOR2/IL23A/IL6/IFITM1/JAK3/FOS/PTPN13/CXCL5/CSF3/IL15/BCL6/MRC1/CXCL11/IL31RA/OSMR/CSF2RB/IFITM3/ZFP36/SNX10/IFITM2/SOD2/EGR1/CXCL3/ACKR |

| | | | | | |
|---|---|---|---|---|---|
| | | | | | 3/GBP2/PARP9/NR5A2/LEPR/PIK3R1/SBNO2/TIMP1/CEACAM1/IL18R1/IL6ST/RAB20/CASP1/HK2/JUNB/PSMB9/CAMK2D/IL17RA/IL6R/CCL2/CDC42EP4/TNFRSF1A/MAP3K8/CEBPB/HMOX1/IFI16/IFI35/PTP4A3/IL18BP/IL15RA/YBX3/ADAM23/CD47/CYBA/ARID5B/FAS/TRAF3IP2/JAK1/ROBO1/FOXO1/MAP3K5/STAT5A/MAPK11/PSMB8/SLC26A6/NMI/EPS8/RNF138/NLRC5/NFKBIA/STAT3/NFIL3/IFNGR1/STAT5B/UGCG/LTBR/WDR35/SIGIRR |
| GO:0019221 | cytokine-mediated signaling pathway | 79 | 79/863 | 4.29E-05 | DUOX1/HGF/CARD14/ACKR1/CIITA/IL1R2/DCST1/CLDN18/IL17RB/CD27/CXCR6/GPR17/CCL16/RHOU/OPRD1/CCL23/CXCL2/IL1R1/SOCS3/IL1RL2/IL33/CEBPD/IL7/IL23A/IL6/IFITM1/JAK3/FOS/CXCL5/CSF3/IL15/BCL6/CXCL11/IL31RA/OSMR/CSF2RB/IFITM3/IFITM2/SOD2/EGR1/CXCL3/ACKR3/GBP2/PARP9/LEPR/PIK3R1/TIMP1/CEACAM1/IL18R1/IL6ST/CASP1/JUNB/PSMB9/CAMK2D/IL17RA/IL6R/CCL2/TNFRSF1A/MAP3K8/HMOX1/IFI35/IL18BP/IL15RA/FAS/TRAF3IP2/JAK1/ROBO1/FOXO1/STAT5A/PSMB8/NMI/NLRC5/NFKBIA/STAT3/IFNGR1/STAT5B/UGCG/LTBR/SIGIRR |

| | | | | | |
|---|---|---|---|---|---|
| GO:0007167 | enzyme linked receptor protein signaling pathway | 87 | 87/863 | 4.29E-05 | HGF/FLRT1/NPPA/PLCE1/SMPD3/KLB/NRG4/TMPRSS6/BMPR1A/CHRNA3/LRG1/IGFBP1/ANKS1B/SOCS3/CSPG4/DCN/INHA/PDGFRA/FLRT3/TMEM100/SHC4/JAK3/FOS/BMPER/ITGB3/AFAP1L2/LCP2/TGFBR3/IL31RA/BDKRB2/ATP6V1C2/SMAD9/OSMR/PCSK6/BMP8A/ADAMTS3/PPM1L/LRRC32/ADRB2/EGR1/DOK5/RGS14/PIK3R1/SPRED3/CEACAM1/IGFBP3/TGFB3/FAT4/PRKCQ/IL6ST/PTPRU/CITED2/SPRY1/FGFR3/CLDN5/SH3TC2/ID1/TXNIP/PTP4A3/SORBS1/FES/MVP/CYBA/DDR2/PIK3C2A/ARID5B/ACVR1B/ROBO1/FOXO1/PSEN2/STAT5A/MAPK11/UBE2D1/MET/STAT3/EFNB1/STAT5B/NIBAN2/LGMN/HHEX/DUSP22/BMPR2/TEK/AP2S1/UBE2D3/TGFBR2/CPNE3 |

**Supplemental Table 7: Detailed results of the top five depleted GO terms (biological processes) obtained from the GO enrichment analysis conducted on the *in vitro* RNA sequencing data OSM+VEGF vs VEGF controls.**

| ID | Description | Count | GeneRatio | padj | geneID |
|---|---|---|---|---|---|
| GO:0000902 | cell morphogenesis | 138 | 138/1221 | 9.11E-14 | GNA12/LIMK1/DAG1/VCL/PAFAH1B1/PTK2/FRMD6/APBB2/RCC2/RANBP9/AR/BIN3/LMTK2/RASA1/FMNL2/PDZD8/MACF1/CAMSAP1/MYADM/TBC1D20/RHOBTB2/NEXN/CDH5/ACTN4/LAMB1/SIPA1L3/PLCG1/MED1/TRIO/FAM171A1/PLXNA3/ARAP1/FZD4/EXT1/HEG1/AFG3L2/RAPGEF2/DNMBP/SKIL/MKLN1/FLNA/RHOB/PDLIM7/MAP1S/NFATC4/MICALL2/SPTAN1/ARHGAP18/KANK1/FGD4/TRAK1/CFAP410/P4HB/SMURF1/DLC1/SDC2/KIF13B/ADARB1/SEMA6D/MYO10/EPHA2/SOX17/VHL/MEF2A/CAP1/RND3/PLXNA4/NTN4/FARP1/ZSWIM8/SPTB |

| | | | | | |
|---|---|---|---|---|---|
| | | | | | N1/MELTF/MYH10/ROBO4/PXN/RHOBTB1/BCL2/RILPL1/ARHGDIA/CDH2/NRK/COL18A1/ZSWIM4/FMNL3/TIAM2/EFNB2/AXL/CDH24/DCHS1/TNFRSF12A/ARHGEF28/FLOT1/GAB1/FLNB/SHROOM3/MERTK/ITGA4/SPTBN5/BCL9L/C12orf57/FGD1/PLXNA2/CPNE5/CAP2/NEDD4L/ZRANB1/COCH/SYT17/WASF3/P2RX7/UNC5B/TPM1/LIMD1/RAB8A/SPINT2/UBB/CXCR4/SYT1/SIAH1/GDF7/EPHB1/NR4A3/SARM1/TUBB2B/SPTA1/MAP2/NOG/SLIT3/NEDD9/FAT1/BDNF/RHOF/RIMS1/ZNF365/KIT/TGFB2/RAPH1/ILK |
| GO:0030029 | actin filament-based process | 108 | 108/1221 | 4.76E-11 | LIMK1/PAFAH1B1/FRMD6/CNN3/FHOD1/ITGB5/HSP90B1/BIN3/PPM1F/AMOTL1/SH3PXD2B/RASA1/ADD3/FMNL2/MYADM/GBA2/MAPKAP1/SSH2/RHOBTB2/ACTN4/MYL6B/FAM171A1/ARAP1/NCK2/ELMO3/PDE4D/MKLN1/FLNA/MYO9B/RHOB/PDLIM7/MYO1D/MICALL2/SPTAN1/CDC42BPB/KPTN/KANK3/FSCN1/PDLIM3/PIP5K1C/PPFIA1/ARHGAP18/PDPK1/ZYX/KANK1/FGD4/PHLDB2/SDC4/CDC42BPA/HIP1R/DLC1/AMOTL2/ATP1A1/KIRREL1/MEF2A/CAP1/RND3/CGNL1/NEB/SPTBN1/DMD/CAV1/CORO6/MYH10/PXN/RHOBTB1/BCL2/SORBS2/ARHGDIA/ANKRD1/NRK/SPIRE1/CELSR1/FMNL3/SUN2/CORO2B/EPB41L4A/GAB1/FLNB/SHROOM3/FRMD5/CAVIN3/MYO5C/SPTBN5/LURAP1/FGD1/MYLK2/CAP2/NEDD4L/WASF3/MICAL2/MTSS1/TPM1/JAM3/RGCC/MYOZ2/VIM/SPTA1/ARPC1B/DAAM2/CX3CL1/NEDD9/FAT1/RHOF/KIT/ELN/RANGRF/ILK |
| GO:0097435 | supramolecular fiber | 102 | 102/1221 | 4.76E-11 | LIMK1/PAFAH1B1/FHOD1/ITGB5/RANBP9/HSP90B1/BIN3/PP |

| | | | | | |
|---|---|---|---|---|---|
| | organization | | | | M1F/SH3PXD2B/RASA1/ADD3/CAMSAP1/MYADM/GBA2/SSH2/RHOBTB2/COLGALT1/CDH5/ACTN4/GOLGA2/NCKAP5L/FAM171A1/ARAP1/SNCA/NCK2/ELMO3/FLNA/RHOB/MAP1S/LOXL2/MYO1D/MICALL2/SPTAN1/KPTN/KANK3/FSCN1/PDLIM3/PPFIA1/LDLR/ARHGAP18/ZYX/KANK1/DYNC1H1/PHLDB2/SDC4/CLIP2/HIP1R/DLC1/KIRREL1/MEF2A/TUBGCP4/CAP1/RND3/COL12A1/CGNL1/PLK3/TTBK2/NEB/SPTBN1/COL5A1/CORO6/MYH10/PXN/RHOBTB1/BCL2/SORBS2/THSD4/ANKRD1/SPIRE1/CORO2B/SHROOM3/MYO5C/NUMA1/SPTBN5/LOXL4/FOXC2/RIPK3/LTBP2/CAP2/WASF3/MICAL2/FIGNL2/TPM1/RGCC/MYOZ2/VIM/SPTA1/ARPC1B/MAP2/CX3CL1/NEDD9/NCKAP5/FAT1/WDR73/COL1A2/COL11A1/RHOF/TRIM54/ELN/TGFB2/RANGRF/ILK |
| GO:0030036 | actin cytoskeleton organization | 97 | 97/1221 | 2.04E-10 | LIMK1/PAFAH1B1/FRMD6/CNN3/FHOD1/ITGB5/HSP90B1/BIN3/PPM1F/AMOTL1/SH3PXD2B/RASA1/ADD3/FMNL2/MYADM/GBA2/MAPKAP1/SSH2/RHOBTB2/ACTN4/FAM171A1/ARAP1/NCK2/ELMO3/MKLN1/FLNA/RHOB/PDLIM7/MYO1D/MICALL2/SPTAN1/CDC42BPB/KPTN/KANK3/FSCN1/PDLIM3/PIP5K1C/PPFIA1/ARHGAP18/PDPK1/ZYX/KANK1/FGD4/PHLDB2/SDC4/CDC42BPA/HIP1R/DLC1/AMOTL2/KIRREL1/MEF2A/CAP1/RND3/CGNL1/NEB/SPTBN1/CORO6/MYH10/PXN/RHOBTB1/BCL2/SORBS2/ARHGDIA/ANKRD1/NRK/SPIRE1/CELSR1/FMNL3/CORO2B/EPB41L4A/GAB1/FLNB/SHROOM3/FRMD5/CAVIN3/MYO5C/SPTBN5/LURAP1/FGD1/CAP2/WASF3/MICAL2/MTSS1/T |

| | | | | | |
|---|---|---|---|---|---|
| | | | | | PM1/JAM3/RGCC/MYOZ2/SPTA1/ARPC1B/DAAM2/CX3CL1/NEDD9/FAT1/RHOF/KIT/ELN/ILK |
| GO:0022603 | regulation of anatomical structure morphogenesis | 138 | 138/1221 | 3.56E-09 | GNA12/LIMK1/MAGED1/DAG1/PAFAH1B1/PTK2/RCC2/AR/HGS/HSPB1/RASA1/FMNL2/PDZD8/MACF1/CAMSAP1/MYADM/RHOBTB2/PKN1/CDH5/ACTN4/CARM1/PLCG1/MED1/FAM171A1/PLXNA3/ARAP1/FZD4/LFNG/RAPGEF2/EMC10/DNMBP/SKIL/MKLN1/FLNA/THBS1/RHOB/NFATC4/COL4A2/EHD1/ARHGAP18/PDPK1/PSMC1/KANK1/FGD4/TRAK1/CFAP410/AKT3/PHLDB2/P4HB/TUG1/SMURF1/DLC1/SDC2/FGFR1/KIF13B/SEMA6D/RRAS/MYO10/EPHA2/PLK2/RND3/PLXNA4/NFATC2/NTN4/SLC12A2/ZSWIM8/COL5A1/MELTF/MYH10/GTF2I/RHOBTB1/BCL2/PHLDB1/TBX1/ARHGDIA/CDH2/ECM1/SPIRE1/ZSWIM4/CELSR1/FMNL3/TIAM2/EFNB2/SMURF2/SPRY2/PSMD7/MEOX2/TNFRSF12A/FLOT1/GAB1/SHROOM3/ROR1/TNFAIP3/JCAD/PGF/BCL9L/FOXC2/ANXA3/FGD1/PLXNA2/CPNE5/NEDD4L/ZRANB1/COCH/SYT17/EPN2/WASF3/HDAC9/TPM1/LIMD1/RGCC/CXCR4/SYT1/GDF7/PPARG/SARM1/TUBB2B/PRICKLE1/VASH1/SERPINE1/SPTA1/ADAM12/MAP2/HSPB6/CX3CL1/NOG/NEDD9/JMJD8/PINK1/PTGIS/BDNF/CAMK1/CD34/RHOF/RIMS1/KIT/TGFB2/ILK |

**Supplemental Table 8: Detailed GSEA results of the top 5 most enriched GO biological processes in the *in vivo* RNA sequencing. Enrichment refers to upregulation in OSM injected samples in contrast to the PBS control.**

| pathway | padj | leading edge |
|---|---|---|

| | | |
|---|---|---|
| GOBP_COTRANSLATIONAL_PROTEIN_TARGETING_TO_MEMBRANE | 0.0001688 9 | RPL22, RPL37, RPS28, RPS29, RPL38, RPL35A, RPS15A, RPL35, RPS24, RPL37A, RPS23, RPL36, RPS13, RPL39, RPLP2, RPL27, RPL23, RPL30, RPL28, RPS21, RPS27, RPL32, RPLP1, SRP19, RPS11, RPL18A, RPL17, RPS14, TRAM1, RPL23A, RPS20, RPS25, SRP68, SRPRA, RPS7, SEC61A2, RPL15, RPL7, RPL10A, RPS19, RPL34, RPL29, RPS16, SSR1, ARL6IP1 |
| GOBP_ESTABLISHMENT_OF_PROTEIN_LOCALIZATION_TO_ENDOPLASMIC_RETICULUM | 3.49E-05 | RPL22, RPL37, RPS28, RPS29, HERPUD1, RPL38, RPL35A, RPS15A, RPL35, RPS24, RPL37A, RPS23, RPL36, RPS13, RPL39, RPLP2, RPL27, RPL23, RPL30, RPL28, RPS21, RPS27, RPL32, RPLP1, SRP19, RPS11, RPL18A, RPL17, RAB10, RPS14, MAN1A1, TRAM1, SEC61G, RPL23A, SPCS3, RPS20, RPS25, SRP68, SRPRA, RPS7, SEC61A2, RPL15, RPL7, RPL10A, RPS19, RPL34, RPL29, RPS16 |
| GOBP_PROTEIN_LOCALIZATION_TO_ENDOPLASMIC_RETICULUM | 0.0014753 | RPL22, RPL37, KDELR1, RPS28, RPS29, HERPUD1, RPL38, RPL35A, RPS15A, RPL35, RPS24, RPL37A, RPS23, RPL36, RPS13, RPL39, RPLP2, RPL27, RPL23, RPL30, RTN4, RPL28, RPS21, RPS27, RPL32, RPLP1, SRP19, RPS11, GPAA1, RPL18A, RPL17, RAB10, RPS14, MAN1A1, TRAM1, SEC16A, SEC61G, RPL23A, SPCS3, RPS20, RPS25, SRP68, SRPRA, RPS7, SEC61A2, RPL15, RPL7, RPL10A, RPS19, RPL34, RPL29, RPS16 |
| GOBP_RESPONSE_TO_TYPE_I_INTERFERON | 0.0016649 3 | IFIT3, ISG20, IFIT1B, ISG15, XAF1, PSMB8, GBP2, IRF7, BST2, STAT1, IFITM1, USP18, RSAD2, IRF9, OASL, ADAR, IFIT2, PTPN2, OAS1, SMPD1, ZBP1, SAMHD1, IFI35, IRF8 |
| GOBP_RESPONSE_TO_VIRUS | 0.0068977 1 | IFIT3, ISG20, TRIM5, IRGM, IFI44, IFIT1B, ISG15, DDX60, ITCH, TKFC, ZC3HAV1, RRP1B, IRF7, RTP4, |

| | | |
|---|---|---|
| | | BST2, STAT1, AGBL5, IFITM1, HMGA1, RSAD2, SIN3A, IL15, IRF9, PYCARD, POLR3E, DHX58, OASL, ADAR, EIF2AK2, IFIT2, IFI16, BNIP3, POLR3H, IKBKG, IFNGR1, SELENOK, RPS15A, BCL3, LYST, OAS1, PLSCR1, POLR3D, CCL19, CCDC130, SMPD1, ZBP1, SAMHD1, IFIH1, PARP9, DDX58, CRCP, PMAIP1 |

**Supplemental Table 9: Detailed GSEA results of the top 5 most depleted GO biological processes in the *in vivo* RNA sequencing. Depletion refers to downregulation in OSM injected samples in contrast to the PBS control.**

| pathway | padj | leading edge |
|---|---|---|
| GOBP_ACTIN_FILAMENT_BASED_PROCESS | 0.00016239 | CORO1A, RGS4, PTK7, HIP1R, ATP1A2, RACGAP1, FAT1, MKKS, CDC42BPB, WIPF1, LRP1, LATS1, CX3CL1, ADD3, AMOTL1, PDGFRB, CCDC88C, SRF, DIAPH3, SPTBN2, CIT, RASA1, MYO18A, GJC1, IKBKB, ANK2, KCNJ8, NF1, IQSEC1, IQGAP3, MTOR, JAK2, SPECC1, ABL1, CFLAR, RAP1GDS1, PHLDB2, TAOK2, PGM5, AQP1, OBSL1, DES, NOS1AP, PPP1R9B, PHACTR1, ACAP2, FGD6, CCDC88A, ARHGEF5, ESPN, MINK1, WASHC2C, PRKCD, SHROOM2, FLNA, SLC9A3R1, STARD8, CLASP2, TLN1, EPDR1, MYH10, EPB41, MRTFA, ELMO2, MET, DIAPH2, ARHGEF17, CDC42BPA, MYH9, NUP155, SRC, ARHGEF15, ANXA1, FHL3, ABITRAM, ARHGAP6, TRPM4, NOX4, PRKCI, CGNL1, ARHGEF11, SHC1, GPD1L, CYRIA, CARMIL1, RTKN, ECT2, ANLN, TSC1, LIMA1, SPTBN1, ROCK1, SPTAN1, ZYX, VIL1, MSRB2, CARMIL2, SLC9A1, ITGB5, ACTA1, GAS2L1, CTTN, ARHGDIB, PIP5K1A, TF, MPRIP, NPHP1, ARHGAP35, STMN1, TRPM7, WASHC5, TRIP10, ARFIP2, SUN2, SRGAP2, ARFGEF1, EMP2, NCKAP1, MYO1F, CYFIP2, NEB, MYO1C, PXN, CORO2B, SHANK3, NCK2, AKAP13, SMTN, RHOBTB2, ARAP1, ACTN1, PDCL3, CCN2, CORO1B, SNX9, INPPL1, FAM171A1, |

| | | |
|---|---|---|
| | | NAA80, IQGAP2, CSF1R, PARVB, NISCH, SCN3B, S100A10, DTNBP1, SLIT2, MYO19, CYRIB, ICAM1, FERMT2, SEMA5A, PAWR, CYFIP1, MYO1D, CYTH2, DAAM1, SELE, CDK5 |
| GOBP_CHROMOSOME_ORGANIZATION | 1.13E-06 | KNSTRN, CDK5RAP2, PRKCB, NCOA1, UBE2C, CCNE2, PRIM2, DSN1, PAXBP1, INCENP, CHD2, NUF2, MKI67, TINF2, RACGAP1, ERCC3, HELLS, FEN1, KDM2B, XRCC5, SMC4, XRCC6, LATS1, MCM8, TERF2, MCPH1, PPM1D, XPC, NSL1, ASF1B, YLPM1, DFFB, MYSM1, ING2, IPO4, HDAC4, CENPF, MRE11, PABPC1L, CDT1, ZBTB7B, HDAC6, NCAPD3, TPR, UHRF1, MCM3AP, CDC45, TNKS, HAT1, UBR2, CDC73, AURKB, SETD7, MCM4, PIBF1, SUPT7L, MCM5, BCOR, KAT6B, DHX36, ARID1A, KIF2C, LRWD1, JAK2, ABL1, MIS18BP1, MCM3, APBB1, BUB1, ZNF830, MAP3K7, PDS5B, PRC1, POLD1, TRIP13, MCRS1, POLE2, MAD2L1, RAD21, KDM5C, HCFC1, BUB1B, IWS1, DNMT1, XPA, MCM7, CENPN, AURKA, FBXL19, KNL1, CHD9, NUP107, PIH1D1, DCLRE1C, PRKCD, RPA1, TFIP11, PCGF3, CHMP7, RIF1, POLA1, EZH2, AIFM1, KAT14, ATXN7L3, XRCC3, KDM1A, KPNB1, UBA52, WRNIP1, PCNA, PBRM1, USP16, LRRK2, CHEK1, ACD, SMARCA4, GRWD1, BRD8, BAHD1, MTERF1, ZMIZ2, BMI1, NUP155, CENPM, TEP1, AKAP8L, NCAPH, SPAG5, MCM9, SRC, ANXA1, H2AX, ACTR5, SMG5, ZNF462, RFC4, KMT2E, ASCC3, PRMT2, PLK1, TWNK, TRIM16, WDR5B, CCNA2, SRCAP, TAF1L, TAF1, TOP2A, NDC80, CTC1, KIF22, TOP2B, OIP5, KDM6B, DDX1, EML4, KDM5B, YEATS2, MRGBP, ERCC6, CBX2, NAV2, SIRT1, PRIM1, MCM2, NASP, NEK6, L3MBTL3, ERCC4, NAA50, HMGN5, FBXO5, PSMG2, H1-5, KDM6A, CENPE, CBX6, NAP1L1, DOT1L, H1-2, CENPP, PRMT7, TCF7L1, LTO1, CENPW, RESF1, HCFC2, RPS6KA5, CENPT, CDC6, SERTAD1, KDM3A, TASOR, GLYR1, ATRX, KDM5A, TRIM28, TADA2A, SMYD3, PHF19, CENPA, WDR5, RBM14, ATXN7, |

| | | |
|---|---|---|
| | | RNF20, ERCC6L, MIER1, ZCWPW1, SMC3, PRKD1, AXIN2, SGF29, SUPT6H, H2AW, MLLT6, CBX8, RFC5, CDC20, SRPK1, REST, PHF14, GEM, CENPS, CABIN1, SMYD2, IGHMBP2, KDM4B, DCLRE1B, BRD4, CDC27, TRAPPC12, NUP133, NCAPG, BRD1, SMARCD1, P3H4, CENPL, NSD2, TAF7, SUDS3, ATM, NUP62, TOP3B, RELA, DDB1, SETD2, ZFPM1, TRRAP, MAP10, MLLT3, HUWE1, SMC6, CHD1, CTR9, TACC3, EXOSC10, VEGFA |
| GOBP_REGULATION_OF_GTPASE_ACTIVITY | 0.00056487 | RGS4, PLXNB2, RACGAP1, MKKS, CX3CL1, DOCK10, RGS5, CPEB2, TBC1D2B, TBC1D30, RASA1, ASAP2, DEPDC5, NF1, IQSEC1, IQGAP3, CHN1, MTOR, PBXIP1, RP2, FAM13B, RAP1GDS1, AGAP3, DOCK1, USP6, ASAP1, OCRL, TSC2, ADAP2, ACAP2, FGD6, ARHGEF5, TIAM2, GPS2, STARD8, TBCD, EZH2, VAV2, PKP4, RASA3, MET, GAPVD1, ARHGAP31, LRRK2, SH3BP4, ERBB2, TBC1D12, PLXNA1, SIPA1L2, ARHGEF15, ARHGAP6, ARHGAP20, ARHGEF11, ELMOD3, ARHGAP11B, ARHGAP11A, RTKN, ECT2, FAM13A, TSC1, ARFGAP1, EVI5, TBC1D10A, MAP4K4, HACD3, DVL2, SEC23B, CHML, IPO5, ARHGAP39, ARHGDIB, RIN1, TBC1D1, PIP5K1A, ARHGAP35, DENND1A, DOCK8, STMN1, SBF2, SRGAP2, AFDN, EPHA2, ARFGEF1, DVL3, TBC1D9B, RABEP2, AGRN, NPRL2, DOCK5, RANBP2, DNM1L, NET1, ARAP1, RASGRP2, TBC1D2, ARFGAP3, F2R, SNX9, WNK1, ARHGEF6, SIPA1L3, IQGAP2, TBC1D17, TBC1D9, ACAP3, S100A10, SLIT2, SEMA4D, KALRN, ICAM1, ARHGAP27, FERMT2 |
| GOBP_CYTOSKELETON_ORGANIZATION | 1.82E-06 | CORO1A, SASS6, KNSTRN, CDK5RAP2, RGS4, PLD2, PTK7, SPC25, INCENP, HIP1R, NUF2, MAP6, RACGAP1, FAT1, MKKS, CDC42BPB, WIPF1, LRP1, LATS1, CEP295, CRYAB, CX3CL1, MCPH1, ZMYM3, ADD3, AMOTL1, DCLK2, PDGFRB, CCDC88C, CAMSAP1, SRF, NES, DIAPH3, CYLD, HDAC6, SPEF1, SPTBN2, |

| | | |
|---|---|---|
| | | TPR, CIT, RASA1, TNKS, KIF11, ODF2, AURKB, CNTLN, MYO18A, IKBKB, PIBF1, KIF2C, NF1, HAUS4, IQSEC1, IQGAP3, MTOR, JAK2, SPECC1, ABL1, CFLAR, GCC2, CCNF, RAP1GDS1, PHLDB2, TAOK2, CNTRL, PGM5, PRC1, CEP135, AQP1, OBSL1, DES, DPYSL2, MAD2L1, NOS1AP, TMEM67, UVRAG, CLIP2, PPP1R9B, IQCG, PHACTR1, KIF24, FGD6, AURKA, CCDC88A, ARHGEF5, ESPN, GADD45A, MINK1, WASHC2C, PRKCD, SHROOM2, FLNA, SLC9A3R1, STARD8, CLASP2, PCNT, TBCD, RCC1, TLN1, MYH10, XRCC3, EPB41, MRTFA, KPNB1, ELMO2, RNF19A, MAST2, HAUS1, MET, DIAPH2, ARHGEF17, CHEK1, CEP192, CDC42BPA, MYH9, PARD3B, GPSM2, E2F4, SPAG5, PLK4, KIF3B, SDCCAG8, SRC, ARHGEF15, ANXA1, FHL3, ABITRAM, ARHGAP6, GAN, MYCBP2, BRWD3, NOX4, PRKCI, ZPR1, PLK1, CGNL1, ARHGEF11, SHC1, EVPL, CEP70, NDC80, CYRIA, ZMYM4, GNAI1, CARMIL1, RTKN, ECT2, ANLN, EML4, CEP250, DYNC1H1, TSC1, NCKIPSD, SIRT1, LIMA1, SPTBN1, ROCK1, SPTAN1, SKA2, NEK6, ZYX, TRAF3IP1, VIL1, FBXO5, MSRB2, CARMIL2, TTLL13P, MARK3, SPRYD3, SLC9A1, CENPE, ITGB5, WTIP, ACTA1, TUBB4A, GAS2L1, PPP1R12A, STIL, CTTN, KIZ, ARHGDIB, FBXW11, CCDC66, DST, PIP5K1A, TF, MPRIP, NPHP1, ARHGAP35, STMN1, TASOR, TRPM7, ATRX, WASHC5, TRIP10, ARFIP2, HOOK3, SUN2, SRGAP2, SRGAP2C, ARFGEF1, WDR62, CENPA, MARK2, RBM14, ATXN7, EMP2, NCKAP1, MYO1F, CYFIP2, SMC3, SYNM, NEB, DAG1, CEP120, CEP350, MAP4, AGRN, MYO1C, RANBP9, PXN, SLK, HAUS5, CORO2B, SHANK3, NCK2, CDC20, AKAP13, CLTC, SMTN, RHOBTB2, TPPP3, TTLL1, ARAP1, ACTN1, PDCL3, USP33, CCN2, MDM1, PAK4, CORO1B, KIF20A, NEXN, SNX9, INPPL1, TTC12, NUP62, SETD2, FAM171A1, NAA80, TUBA1C, SIPA1L3, MAP10, IQGAP2, |

| | | |
|---|---|---|
| | | CSF1R, NCKAP5L, TACC3, TUBA4A, MAP1A, PARVB, NISCH, OCLN, KATNAL1, S100A10, CUL7, DTNBP1, SLIT2, MYO19, TNFAIP3, NIN, CYRIB, ICAM1, FERMT2, DLGAP5, INTS13, SEMA5A, PAWR, CKAP5, CYFIP1, MYO1D, CYTH2, DAAM1, KIF4A, CDK5 |
| GOBP_MITOTIC_CELL_CYCLE | 7.90E-05 | KNSTRN, CDK5RAP2, PRKCB, SKP2, UBE2C, CCNE2, PPAT, LZTS1, SPC25, PRIM2, DSN1, INCENP, NUF2, MKI67, RACGAP1, ERCC3, DHFR, SMC4, LATS1, MCM8, MCPH1, PPM1D, XPC, NSL1, WDHD1, CDKN3, PDGFRB, NEK3, CENPF, MRE11, CDT1, FZD3, CTDSPL, OFD1, NES, CYLD, NCAPD3, TPR, MDM2, CIT, RASA1, CDC45, TNKS, TIMP2, KIF11, CDC73, ODF2, E2F1, AURKB, MCM4, PIBF1, MCM5, ANKLE2, KIF2C, HAUS4, IQGAP3, ABL1, MCM3, ADAM17, RRM2, CCNF, BUB1, TAOK2, CNTRL, ZNF830, PDS5B, PRC1, CDKN2B, CEP135, OBSL1, TFDP2, TRIP13, MUS81, POLE2, MAD2L1, RAD21, PPP1R9B, NPAT, BUB1B, HYAL1, MCM7, AURKA, RRM1, NUP214, LIG1, GADD45A, RPA1, FLNA, SLC9A3R1, CHMP7, ADAMTS1, CLASP2, POLA1, PCNT, TAOK3, TBCD, RCC1, EZH2, MYH10, XRCC3, KPNB1, HAUS1, CEP290, PCNA, PBRM1, USP16, PBK, CHEK1, CEP192, ASAH2, GPSM2, E2F4, MRNIP, AKAP8L, NCAPH, SPAG5, PLK4, KIF3B, SDCCAG8, ANXA1, MASTL, KMT2E, FBXO31, ZPR1, PRMT2, LPIN1, PLK1, ZFYVE19, PHLDA1, CCNA2, SFI1, CEP70, NDC80, CTC1, GNAI1, KIF22, RTKN, CDK6, CUL5, ECT2, CTDP1, ANLN, EML4, CEP250, DYNC1H1, CLSPN, SIRT1, SPTBN1, PRIM1, ROCK1, MCM2, NASP, ACTR1A, SKA2, NEK6, DONSON, NAA50, FBXO5, CNOT10, PSMG2, APPL1, CDH13, CENPE, TUBB4A, KLHDC8B, PPP1R12A, STIL, CENPW, PRCC, FBXW11 |

**Supplemental Table 10: Detailed GSEA results of the top 5 most enriched Reactome pathways in the *in vivo* RNA sequencing. Enrichment refers to upregulation in OSM injected samples in contrast to the PBS control.**

| pathway | padj | leading edge |
|---|---|---|
| REACTOME_CELLULAR_RESPONSE_TO_STARVATION | 2.66E-06 | BMT2, RRAGA, RPL22, RPL37, FNIP1, RPS28, RPS29, RPL3L, RPL38, CASTOR2, TRIB3, RPL35A, RPTOR, RPS15A, RPL35, RPS24, RPL37A, RPS23, ATP6V0C, MIOS, RPL36, RPS13, RPL39, RPLP2, RPL27, RPL23, RPL30, SESN2, RPL28, FAU, RPS21, KPTN, RPS27, RPL32, RPLP1, RPS11, SLC38A9, LAMTOR4, RPL18A, RPL17, LAMTOR3, ATF4, DDIT3, EIF2S2, RPS14, RRAGB, RPL23A, RPS20, RPS25, RPS7, RPL22L1, RPL15, RPL7, ATP6V1D, RPL10A, ATP6V0E1, RPS19, RPL34, RPL29, RPS16, SZT2 |
| REACTOME_EUKARYOTIC_TRANSLATION_ELONGATION | 1.52E-08 | RPL22, RPL37, RPS28, RPS29, RPL3L, RPL38, RPL35A, RPS15A, RPL35, RPS24, RPL37A, RPS23, RPL36, RPS13, RPL39, RPLP2, RPL27, RPL23, RPL30, RPL28, FAU, RPS21, RPS27, RPL32, RPLP1, RPS11, RPL18A, RPL17, RPS14, RPL23A, RPS20, RPS25, RPS7, RPL22L1, RPL15, RPL7, RPL10A, RPS19, RPL34, RPL29, RPS16 |
| REACTOME_REGULATION_OF_EXPRESSION_OF_SLITS_AND_ROBOS | 0.0001400968 | PSMB8, RPL22, RPL37, RPS28, PSMB7, RPS29, RPL3L, RPL38, PSMB9, MAGOH, PSMB10, RPL35A, RPS15A, PSMF1, RPL35, RPS24, RPL37A, RPS23, RPL36, RPS13, RPL39, RPLP2, RPL27, RPL23, RPL30, RPL28, FAU, RPS21, RPS27, RPL32, RPLP1, RPS11, PSMA6, RPL18A, RPL17, RPS14, PSMA5, PSMB4, PSMA8, RPL23A, RPS20, RPS25, RPS7, RPL22L1, RPL15, RPL7, PSMB2, RPL10A, RPS19, RPL34, RPL29, RPS16, RNPS1, PSMD5, PSMD10 |
| REACTOME_RESPONSE_OF_EIF2AK4_GCN2_TO_AMINO_ACID_DEFICIENCY | 1.70E-07 | RPL22, RPL37, RPS28, RPS29, RPL3L, RPL38, TRIB3, RPL35A, RPS15A, RPL35, RPS24, RPL37A, RPS23, RPL36, RPS13, RPL39, RPLP2, RPL27, RPL23, RPL30, RPL28, FAU, RPS21, RPS27, RPL32, RPLP1, RPS11, RPL18A, RPL17, ATF4, DDIT3, EIF2S2, RPS14, RPL23A, RPS20, RPS25, RPS7, RPL22L1, RPL15, RPL7, RPL10A, RPS19, RPL34, RPL29, RPS16 |
| REACTOME_SRP_DEPENDENT_COTRANSLATIONAL_PROTEIN_TARGETING_TO_MEMBRANE | 8.12E-10 | RPL22, RPL37, RPS28, RPS29, RPL3L, RPL38, RPL35A, RPS15A, RPL35, RPS24, RPL37A, RPS23, RPL36, RPS13, RPL39, RPLP2, RPL27, SEC11A, RPL23, RPL30, RPL28, FAU, RPS21, RPS27, RPL32, RPLP1, SRP19, RPS11, RPL18A, RPL17, RPS14, RPN1, TRAM1, SEC61G, RPL23A, SPCS3, RPS20, RPS25, SRP68, SRPRA, RPS7, SEC61A2, RPL22L1, RPL15, RPL7, RPL10A, RPS19, RPL34, RPL29, RPS16, SSR4 |

**Supplemental Table 11: Detailed GSEA results of the top 5 depleted Reactome pathways in the *in vivo* RNA sequencing. Depletion refers to downregulation in OSM injected samples in contrast to the PBS control.**

| pathway | padj | leading edge |
|---|---|---|
| REACTOME_CELL_CYCLE | 5.58E-06 | CDK5RAP2, PRKCB, GINS2, SKP2, UBE2C, CCNE2, SPC25, PRIM2, DSN1, INCENP, LMNB1, NUF2, TINF2, FEN1, DHFR, SMC4, MCM8, TERF2, MCPH1, NSL1, NUP58, CENPF, MRE11, CDT1, OFD1, LIN9, NCAPD3, TPR, MDM2, TK1, CDC45, ODF2, E2F1, AURKB, MCM4, MCM5, ANKLE2, KIF2C, HAUS4, NUP160, JAK2, ABL1, USO1, MIS18BP1, MCM3, NUP205, RRM2, BUB1, CNTRL, PDS5B, CDKN2B, POLD1, CEP135, TFDP2, SPC24, POLE2, MAD2L1, PPP2R5D, RAD21, CC2D1B, BUB1B, MCM7, CENPN, AURKA, NUP214, LIG1, KNL1, NUP107, ATRIP, RPA1, CHMP7, NUP188, CLASP2, POLA1, PCNT, RCC1, KPNB1, UBA52, HAUS1, CEP290, PCNA, CHEK1, CEP192, ACD, POLR2A, E2F4, NUP155, CENPM, NCAPH, PLK4, SDCCAG8, SRC, H2AX, MASTL, RFC4, SYNE1, LPIN1, PLK1, UBE2V2, PHLDA1, CCNA2, TOP2A, SFI1, CEP70, NDC80, CTC1, OIP5, CDK6, RAD1, EML4, CEP250, DYNC1H1, CLSPN, PRIM1, MCM2, NUP42, ACTR1A, SKA2, PMF1, NEK6, FBXO5, CENPE, TUBB4A, CENPP, PPP1R12A, CENPW, FBXW11, CENPT, BIRC5, CDC6, ATRX, VRK2, SUN2, UBE2S, ANKRD28, CENPA, YWHAG, ERCC6L, TYMS, SMC3, CDKN2C, HAUS5, RFC5, NUP35, CDC20, PPP6R3, E2F3, RANBP2, CENPS, GTSE1, CDC27, DYNC1I2, NUP133, KIF20A, NCAPG, CENPL, NSD2, ATM, NUP62 |
| REACTOME_CELL_CYCLE _MITOTIC | 7.58E-07 | CDK5RAP2, PRKCB, GINS2, SKP2, UBE2C, CCNE2, SPC25, PRIM2, DSN1, INCENP, LMNB1, NUF2, FEN1, DHFR, SMC4, MCM8, MCPH1, NSL1, NUP58, CENPF, CDT1, OFD1, LIN9, NCAPD3, TPR, TK1, CDC45, ODF2, E2F1, AURKB, MCM4, MCM5, ANKLE2, KIF2C, HAUS4, NUP160, JAK2, ABL1, USO1, MCM3, NUP205, RRM2, BUB1, CNTRL, PDS5B, CDKN2B, POLD1, CEP135, TFDP2, SPC24, POLE2, MAD2L1, PPP2R5D, RAD21, CC2D1B, BUB1B, MCM7, CENPN, AURKA, NUP214, LIG1, KNL1, NUP107, RPA1, CHMP7, NUP188, CLASP2, POLA1, PCNT, RCC1, KPNB1, UBA52, HAUS1, |

| | | |
|---|---|---|
| | | CEP290, PCNA, CEP192, E2F4, NUP155, CENPM, NCAPH, PLK4, SDCCAG8, SRC, H2AX, MASTL, RFC4, LPIN1, PLK1, PHLDA1, CCNA2, TOP2A, SFI1, CEP70, NDC80, CDK6, EML4, CEP250, DYNC1H1, PRIM1, MCM2, NUP42, ACTR1A, SKA2, PMF1, NEK6, FBXO5, CENPE, TUBB4A, CENPP, PPP1R12A, FBXW11, CENPT, BIRC5, CDC6, VRK2, UBE2S, CENPA, YWHAG, ERCC6L, TYMS, SMC3, CDKN2C, HAUS5, RFC5, NUP35, CDC20, E2F3, RANBP2, CENPS, GTSE1, CDC27, DYNC1I2, NUP133, KIF20A, NCAPG |
| REACTOME_METALLOTHIONEINS_BIND_METALS | 1.04E-06 | MT1G, MT1H, MT1X, MT1F, MT1B, MT1A, MT1M, MT1E, MT2A |
| REACTOME_RHO_GTPASE_EFFECTORS | 7.18E-06 | PRKCB, SPC25, DSN1, INCENP, NUF2, WIPF1, NSL1, CENPF, SRF, DIAPH3, PPP1R14A, CIT, AURKB, KIF2C, IQGAP3, NUP160, ABL1, BUB1, PRC1, MYLK, SPC24, MAD2L1, PPP2R5D, PKN3, NCF2, BUB1B, CENPN, KNL1, NUP107, KLC2, PRKCD, FLNA, CLASP2, MYH10, KDM1A, MRTFA, MYL9, DIAPH2, MYH9, CENPM, SRC, H2AX, MAPK11, PLK1, NDC80, KLC4, RTKN, DYNC1H1, NCKIPSD, ROCK1, SKA2, PMF1, DVL2, CENPE, TUBB4A, CENPP, PPP1R12A, CTTN, CENPT, BIRC5, SRGAP2, KIF5B, CENPA, DVL3, YWHAG, ERCC6L, NCKAP1, CYFIP2, CDC20, RANBP2, CENPS, NCF1, DYNC1I2, NUP133, PIK3R4, CENPL, TUBA1C, IQGAP2, H2BC8, TUBA4A |
| REACTOME_SIGNALING_BY_RHO_GTPASES_MIRO_GTPASES_AND_RHOBTB3 | 5.28E-08 | PRKCB, PLD2, SENP1, SPC25, ZAP70, DSN1, INCENP, LMNB1, RBBP6, NUF2, RACGAP1, CDC42BPB, WIPF1, DOCK10, ADD3, NSL1, MSI2, CENPF, DBT, SRF, DIAPH3, SHKBP1, PPP1R14A, CIT, BASP1, AURKB, ANKLE2, KIF2C, IQGAP3, CHN1, NUP160, STAM2, PDE5A, ABL1, BUB1, FAM13B, RAP1GDS1, PRC1, MYLK, SPC24, DOCK1, MAD2L1, PPP2R5D, PKN3, NCF2, OCRL, BUB1B, WDR91, DLG5, CENPN, CCDC88A, ARHGEF5, DEF6, TIAM2, KNL1, NUP107, KLC2, PRKCD, FLNA, STARD8, CLASP2, TAOK3, VAV2, MYH10, PKP4, TJP2, KDM1A, MRTFA, ELMO2, ABCD3, LETM1, PLIN3, ARHGAP31, MYL9, DIAPH2, ARHGEF17, CDC42BPA, MYH9, WWP2, JAG1, CENPM, PLXNA1, SRC, ARHGEF15, H2AX, TRIO, EFHD2, ARHGAP6, MCF2L, MAPK11, PLK1, ARHGAP20, ARHGEF11, NDC80, KLC4, ARHGAP11B, ARHGAP11A, ZNF512B, RTKN, ECT2, ANLN, FAM13A, DYNC1H1, SOS2, UACA, NCKIPSD, SPTBN1, ROCK1, SPTAN1, SKA2, PMF1, |

| | | |
|---|---|---|
| | | TRAK2, DVL2, CENPE, ARHGAP39, TUBB4A, CENPP, PPP1R12A, RAB9A, SHMT2, CTTN, SLC4A7, ARHGDIB, DST, CENPT, BIRC5, ARHGAP35, DOCK8, C1QBP, ANKFY1, VRK2, TRIP10, SRGAP2, KIF5B, EPHA2, VANGL1, CENPA, DVL3, MPP7, FNBP1, YWHAG, RNF20, ERCC6L, NCKAP1, CYFIP2, MFN2, CAVIN1, SLK, NCK2, CDC20, AKAP13, CLTC, DOCK5, RHOBTB2, RANBP2, NET1, ARAP1, ACTN1, CENPS, ARFGAP3, PAK4, NCF1, SPEN, DYNC1I2, NUP133, PIK3R4, CENPL, PLEKHG1, TUBA1C, ARHGEF6, IQGAP2 |
